# Predicting Cadmium and Arsenic Accumulation and Soil-Exposure Health Risks in Agricultural Soils Below the Risk Screening Values: A Refined Flux Balance Model

**DOI:** 10.3390/toxics14080652

**Published:** 2026-07-24

**Authors:** Tingting Fan, Feiyang Xia, Da Ding, Xiang Wang, Tao Long, Shaopo Deng, Lingya Kong

**Affiliations:** 1 Nanjing Institute of Environmental Science, Ministry of Ecology and Environment, Nanjing 210042, China; bluebird3602@126.com (T.F.); feiyangxia28@163.com (F.X.); dingda@nies.org (D.D.); wangxiang@nies.org (X.W.); longtao@nies.org (T.L.); 2 Key Laboratory of Soil Environmental Management and Pollution Control, Ministry of Ecology and Environment, Nanjing 210042, China

**Keywords:** potentially toxic elements, mass balance model, suspended particulate matter, grain–straw allocation, Ningxia Yellow River irrigation area

## Abstract

Less attention has been paid to soils with potentially toxic elements (PTEs) below agricultural land risk screening values, even though they continue to accumulate these elements. In this study, four typical areas in Ningxia were selected to determine the concentrations of cadmium (Cd) and arsenic (As) in 176 samples collected from 130 sampling sites across seven matrices. A refined mass balance model was developed by partitioning irrigation input into suspended-solid and supernatant phases and crop removal into grain and straw components. The model was used to analyze the contributions of various input and output factors and to predict future soil Cd/As concentrations and their related health risks via soil exposure. The input fluxes of Cd and As in the four regions ranged from 1.31 to 3.25 g·ha^−1^·yr^−1^ and 71.43 to 146.99 g·ha^−1^·yr^−1^, respectively, mainly contributed by irrigation water (40~64%), especially suspended solids in irrigation water, and atmospheric deposition (23~40%). The output fluxes of Cd and As were 0.89~1.43 g·ha^−1^·yr^−1^ and 13.81~33.86 g·ha^−1^·yr^−1^, respectively, dominated by crop harvesting (36~82%). The differences in input and output fluxes were mainly caused by the regional industrial structure and agricultural planting structure. The predicted results showed that soil Cd and As concentrations in all regions would not exceed regulatory limits after 100 years in the current scenario. A health risk assessment based on soil ingestion, dermal contact, and inhalation showed that the hazard indices for Cd and As were negligible, but their total carcinogenic risk reached notable levels. Over time, Cd-specific carcinogenic risk for children increased in several scenarios and transitioned from negligible to notable risk, with soil ingestion being the dominant exposure pathway. According to the results, targeted mitigation strategies, including the regulation of atmospheric deposition, optimization of irrigation water quality, and adoption of straw off-field practices, show potential to effectively limit the accumulation of potentially toxic elements in agricultural soils.

## 1. Introduction

Potentially toxic elements (PTEs), which are naturally occurring elements, are ubiquitous in the environment. With the intensification of human activities, they are continuously released into the environment, particularly in soils. Soil pollution occurs when PTEs concentrations in soil exceed ecological, agricultural, or human health risk thresholds. In total, 14~17% of the global arable land (approximately 242 million hectares) has been contaminated by at least one toxic metal (such as arsenic, cadmium, cobalt, chromium, copper, nickel, or lead), exceeding the agricultural safety threshold [1]. Among these elements, cadmium (Cd) and arsenic (As) warrant urgent attention due to their high mobility in the soil–rice system and subsequent risks to the human food chain [2]. According to the Report on the National General Survey of Soil Contamination, jointly issued by the Chinese Ministries of Environmental Protection and Land Resources [3], Cd and As are among the most severe soil contaminants in China, with non-compliance rates reaching 7.0% and 2.7%, respectively. Consequently, extensive research has focused on their spatiotemporal distribution, source identification, and remediation strategies in heavily polluted regions [4,5,6].

As reported by the United Nations Environment Programme [7], the concentration of PTEs in soil is anticipated to continue rising, given the multiple applications of PTEs, their extraction, use, and global diffusion. Therefore, more attention should also be paid to soils that remain below agricultural land risk screening values. Farmland soils, well known as a sink for PTEs, have a wide range of sources for PTEs [8]. As one of the world’s largest industrial producers, China experiences elevated regional emissions of As and Cd [2]. Zhang et al. [9] found that soil arsenic contamination in China has been deteriorating persistently over the past two decades, with the national mean concentration increasing by approximately 6% from 2000 to 2020, and a further rise of 8% is projected by 2040. A meta-analysis of heavy metal(loid) input fluxes to Chinese agricultural soils (2000–2021) reveals that Cd is the most critical threat to Chinese agricultural soils, with atmospheric deposition as its primary source. At current input rates, soil Cd will exceed safety limits within ~50 years in key farming regions [10]. The major Cd and As input pathways in agricultural soil are atmospheric deposition, irrigation, chemical fertilizer, and other agricultural additives [11]. Without timely prevention and mitigation strategies, continuous inputs from these various sources will inevitably cause PTE concentrations in currently farmlands to exceed safety warning limits.

Flux balance models have been utilized for source apportionment and long-term accumulation forecasting [12,13]. However, these applications have predominantly targeted regions with known or severe contamination, while soils below agricultural land risk screening values, which are critical for proactive risk interception, remain comparatively underexplored. As a dynamic tool, the flux balance model quantifies principal input–output fluxes and simulates future concentration trajectories, providing a scientific basis for source-level control policies. Previous mass balance/flux balance studies [6,13,14,15] have represented irrigation as a single, undifferentiated bulk-water flux and crop output as a single aggregated harvest term, typically without distinguishing between supernatant and suspended solids in irrigation inputs or grain versus straw removal. Because uncontaminated or slightly contaminated soils lack a dominant flux pathway, proactive management requires comparable resolution across all flux pathways, each of which corresponds to an actionable policy lever (e.g., irrigation water quality and crop management practices).

This challenge is particularly acute for irrigation in systems where rivers carry high suspended sediment loads, a complexity that existing flux balance models rarely account for, as such sediments can carry substantial quantities of particulate-bound metal(loid)s. This study, therefore, selected the Ningxia Yellow River Irrigation District as a representative case to address this gap. The Yellow River Basin (YRB) serves as both a core grain-production region and a major industrial base for energy, chemicals, and raw materials in China. Ningxia, situated in the upper reaches of the YRB, is undergoing an intermediate stage of industrialization dominated by resource-, energy-, and heavy chemical-based industries [16], and its farmland is irrigated predominantly with Yellow River water characterized by extremely high suspended sediment concentrations capable of adsorbing and transporting industrial pollutants from upstream sources [17].

In the Ningxia Yellow River irrigation area, the concentrations of heavy metals in agricultural soils are all safely below China’s regulatory risk screening values; however, they exhibit varying degrees of historical enrichment [18,19]. Therefore, proactively predicting the temporal accumulation and identifying the sources of Cd and As in these soils that remain below the corresponding screening value of the Soil Environmental Quality Risk Control Standard for Soil Contamination of Agricultural Land (GB 15618-2018) [20] is imperative for long-term risk management. In this study, four typical areas below agricultural land risk screening values in the irrigation areas along the Yellow River in Ningxia were selected. A refined input–output flux model was developed, which explicitly distinguishes suspended sediment-bound from dissolved metal(loid) inputs via irrigation and partitions crop uptake output between grain and straw. Using this model, this study aims to (1) identify the sources of soil Cd and As, and (2) predict their future concentrations and human health risks via soil exposure. This work provides a scientific foundation for early-warning management and heavy metal pollution prevention in typical arid, high-sediment river irrigation agroecosystems.

## 2. Materials and Methods

### 2.1. Study Area

**Figure 1:**
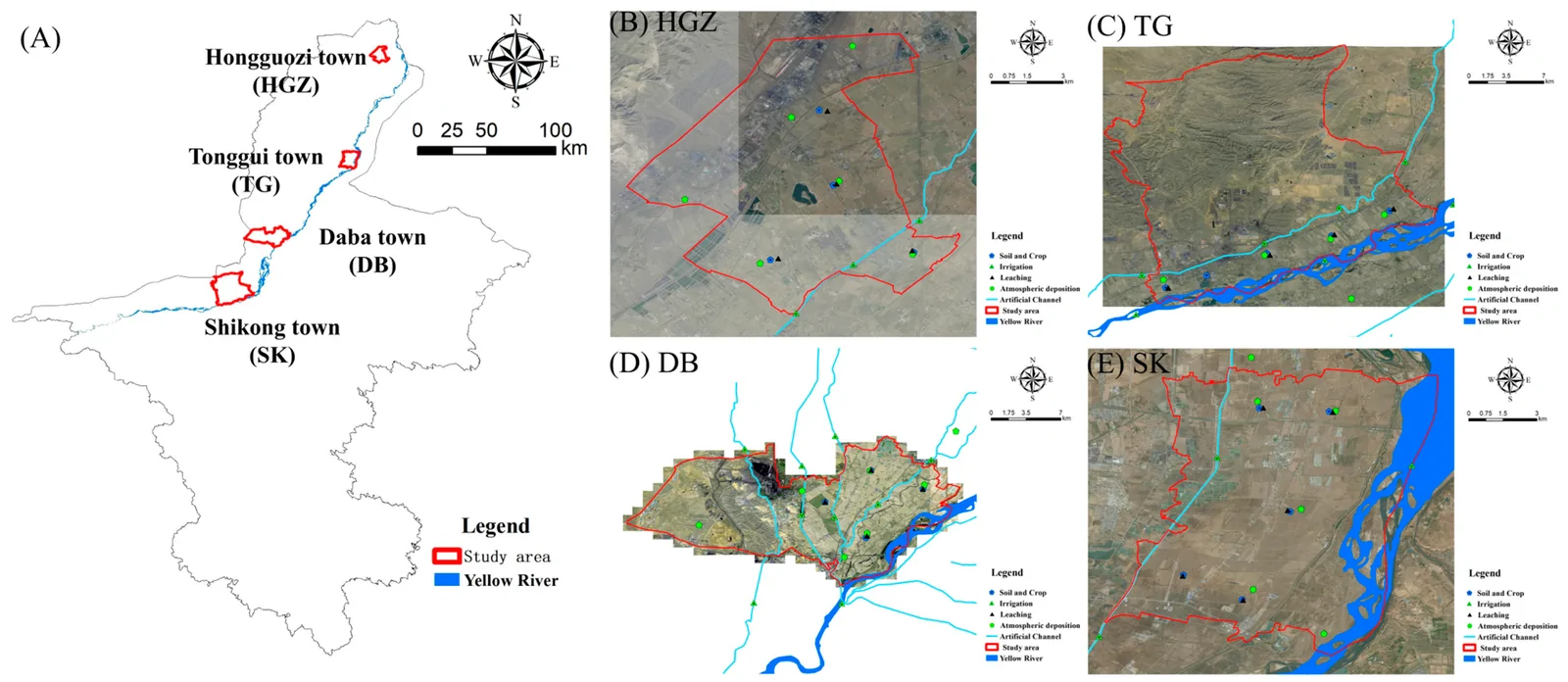
Sampling locations of total atmospheric deposition, irrigation water, leaching, soil, and crop in Ningxia, China. (**A**) Distribution of study areas; (**B**) Hongguozi town (HGZ); (**C**) Tonggui town (TG); (**D**) Daba town (DB); (**E**) Shikong town (SK).

Four distinct functional zones (Figure 1) were selected to predict Cd and As concentrations and soil-exposure health risks. These zones are situated along the Yellow River, each with unique industrial characteristics. Hongguozi town (HGZ) is predominantly an industrial area focused on coal mining, while Shikong town (SK) is known for its nonferrous metal smelting industry. Daba town (DB) represents a mixed industrial area dominated by ferroalloy smelting and nonferrous metal smelting, and Tonggui town (TG) is primarily focused on agricultural development.

Each of these regions experiences a single cropping cycle per year, which includes rice, maize, and wheat. The irrigation period of the Yellow River canals lasts from May to November, with crop-specific watering schedules: maize is irrigated twice, at the end of May and the end of July; wheat receives one irrigation in May during the green-up stage; and rice is irrigated more frequently from May to July. The main agricultural inputs in these areas include compound fertilizers and urea. Sampling was designed to represent local farming practices and the major Cd and As input–output pathways.

### 2.2. Sampling

Sampling sites were set based on preliminary data review; field reconnaissance; interviews with local farmers, irrigation managers and agricultural authorities; land-use patterns; crop type; irrigation systems; and potential industrial emission sources.

#### 2.2.1. Atmospheric Deposition

Atmospheric deposition sampling was conducted in accordance with Specification of Regional Ecogeochemistry Assessment (DZ/T 0289-2015) [21]. The monitoring sites were categorized into three types based on their spatial relationship with pollution sources and dominant wind direction: the first type was located near factories with Cd and As emissions (gas-related pollution source sites), the second type was distributed around farmland to monitor atmospheric input flux, and the third type was designated as reference (clean control) sites unaffected by industrial or agricultural emissions. Dust collectors with an inner diameter of 15 ± 0.5 cm were installed at a height of 5–10 m above ground and, prior to deployment, were soaked in 10% HCl (Sinopharm Chemical Reagent Co., Ltd., Shanghai, China) for 24 h and thoroughly rinsed with distilled water to eliminate potential contamination. A total of 26 atmospheric deposition samples, comprising both dry and wet deposition, were collected from the four study areas using samplers that had been placed in June 2023 and subsequently retrieved in October 2023.

#### 2.2.2. Irrigation Water

To evaluate the influx of Cd and As into farmland via irrigation, water samples were collected from both natural rivers and artificial channels. Based on the distribution of the irrigation system, samples were taken at strategic points: the entrance, middle, and outlets of the irrigation system. Two 500-mL water samples were collected using polyethylene bottles pre-rinsed with site water (2~3 times), with continuous shaking during filling to ensure representativeness. One sample was refrigerated for the determination of suspended solids. The other sample was filtered through a 0.45 μm membrane (Tianjin Jinteng Experiment Equipment Co. Ltd., Tianjing, China) to obtain the filtered supernatant, which was then acidified and refrigerated for Cd and As determination. A total of 42 irrigation water samples were collected at 23 sampling sites twice during the irrigation period in June 2023 and August 2023. No irrigation water samples could be collected at some sites due to the absence of irrigation water.

#### 2.2.3. Sediments

Sediment samples from natural rivers and artificial channels used for irrigation were collected and analyzed to assess the Cd and As flux of suspended solids in irrigation water. The sampling locations for these sediments were identical to those used for collecting irrigation water. Surface sediment samples (0–20 cm) were collected with a spade, cleared of gravel and weeds, homogenized, and 1 kg was sealed in a polyethylene container and refrigerated. Finally, a total of 19 samples were gathered in June 2023, owing to the absence of sediment at several sites.

#### 2.2.4. Fertilizer

Fertilizer sampling followed local farmers’ conventional fertilization practices at each sampling site. A 1 kg fertilizer sample was collected with a sampling spade and then placed in a sealed polyethylene container for storage. During the farmers’ fertilization in June 2023, a total of 9 samples of fertilizers, including compound fertilizer and urea, were collected from these study areas.

#### 2.2.5. Crop Uptake

Separate portions of crops, including grain and straw, were collected for analysis. Within each sampling zone, three 1 m^2^ subplots were set up. A composite sample was formed by combining equal numbers of panicles (10–20 for rice/wheat) or individual plants (3–5 for maize/soybean) collected from each subplot. Straw portions were sampled independently, and each composite sample weighed at least 0.5 kg fresh weight. A total of 36 crop samples, comprising paired grain and straw samples from 18 sampling sites, were collected separately at harvest in September 2023.

#### 2.2.6. Soils

In September 2023, 18 surface soil samples (0–20 cm) were collected using the five-point sampling method. These samples were taken at the same location as the crop sampling site in four study areas (Table S1). Surface soil samples were collected using a stainless steel spade. After removing gravel, plant roots, and other debris, the subsamples collected from multiple points were homogenized, reduced by the quartering method, and placed in polyethylene bags, with a final sample weight of no less than 1 kg.

#### 2.2.7. Leaching and Surface Runoff

The leaching sampling points were strategically positioned to avoid roads, villages, and pollution sources. The leaching samples were collected from a depth of 30~50 cm using custom-built, zero-tension PVC lysimeters that were vertically deployed in 1-m boreholes. To ensure sampling integrity, the screening section (30~60 cm) was wrapped in nylon mesh and packed with quartz stones, while the upper layer was sealed with bentonite alongside a surface-protruding pipe to completely block surface water intrusion. The accumulated subsurface leachate was retrieved, transferred immediately into pre-cleaned polyethylene bottles, acidified, and stored in a portable refrigerator at 4 °C prior to laboratory analysis. A total of 26 effective leachate samples from 17 sampling sites were collected twice during the irrigation period in June 2023 and August 2023, owing to the absence of water in some collectors at specific sampling times.

Because of high evaporation and limited runoff generation in the study areas, surface runoff samples were not available.

### 2.3. Sample Treatment, Analytical and QA/QC

#### 2.3.1. Sample Treatment

The soil samples were air-dried and ground to pass through a 0.15 mm nylon sieve for the analysis of Cd and As concentration in soils. The methods for analyzing PTEs in sediments are analogous to those employed in soils.

Atmospheric mixed deposition samples were air-dried and weighed for dustfall flux determination, followed by digestion and filtration through 0.45 μm membrane filters for Cd and As analysis.

Samples of compound fertilizer and urea were digested in a mixture of HNO_3_/HCl (1:3, *v*/*v*) (Sinopharm Chemical Reagent Co., Ltd., Shanghai, China) according to Determination of arsenic, cadmium, chromium, lead and mercury contents for fertilizers (GB/T 23349-2020) [22] to determine total Cd and As concentrations.

Crop samples were initially rinsed with deionized water, then dried in an oven (Shanghai Jinghong Experimental Equipment Co., Ltd., Shanghai, China) at 105 °C for 2 h, followed by 60 °C for approximately 48 h. Subsequently, the samples were ground into a powder. The plant powders were digested in a mixture of HNO_3_/HClO_4_ (85:15%, *v*/*v*) (Sinopharm Chemical Reagent Co., Ltd., Shanghai, China) to determine total Cd and As concentrations.

Irrigation water was separated into supernatant and suspended solids. When collecting the suspended fraction, the sample bottle was continuously agitated during filling to minimize particle settling and ensure representativeness. Suspended solids in irrigation water were isolated by filtration through 0.45 µm membrane filters following homogenization, with the solids retained on the filters subsequently dried and weighed according to Water quality: Determination of suspended substance: Gravimetric method (GB 11901-1989) [23]. Another 500 mL irrigation sample was filtered through a 0.45 µm membrane to obtain the filtered supernatant, and then acidified and refrigerated for the analysis of Cd and As according to Water and Wastewater Monitoring and Analysis Methods (4th Edition, Supplement) [24] and Water quality: Determination of mercury, arsenic, selenium, bismuth and antimony: Atomic fluorescence spectrometry (HJ 694-2014) [25], respectively.

Cd and As in leachate samples were analyzed using the same procedure as that used for irrigation supernatant samples.

#### 2.3.2. Analytical Methods

All sample collection and analysis were conducted by a third-party commercial laboratory holding China Metrology Accreditation (CMA) certification in Ningxia in accordance with national technical specifications. The analytical methods, detection limits, and other pertinent parameters for different matrices and target analytes are provided in Table S2.

#### 2.3.3. QA/QC

To ensure the representativeness, integrity, comparability, precision, and accuracy of the analytical data, this study strictly adhered to national and local environmental monitoring technical specifications and standard analytical methods throughout the entire workflow, including sampling site design, sample collection, transport and preservation, laboratory analysis, and data processing, with full-process quality assurance and quality control (QA/QC) measures implemented. Specifically, all instruments and equipment were verified or calibrated by accredited metrology authorities and used within their valid calibration periods; all sampling and analytical personnel were appropriately certified for their assigned tasks; sampling locations and frequency were rationally established in strict accordance with testing plan and relevant technical guidelines; and a comprehensive suite of QC procedures was employed, including full-program blanks, laboratory blanks, duplicate analyses, matrix spike recoveries, and certified reference material assays for soil, irrigation water, leachate and sediment. All QC results fell within acceptable control limits, demonstrating compliance with the required criteria. A QC summary is presented in Table S3.

### 2.4. A Refined Flux Balance Model

**Figure 2:**
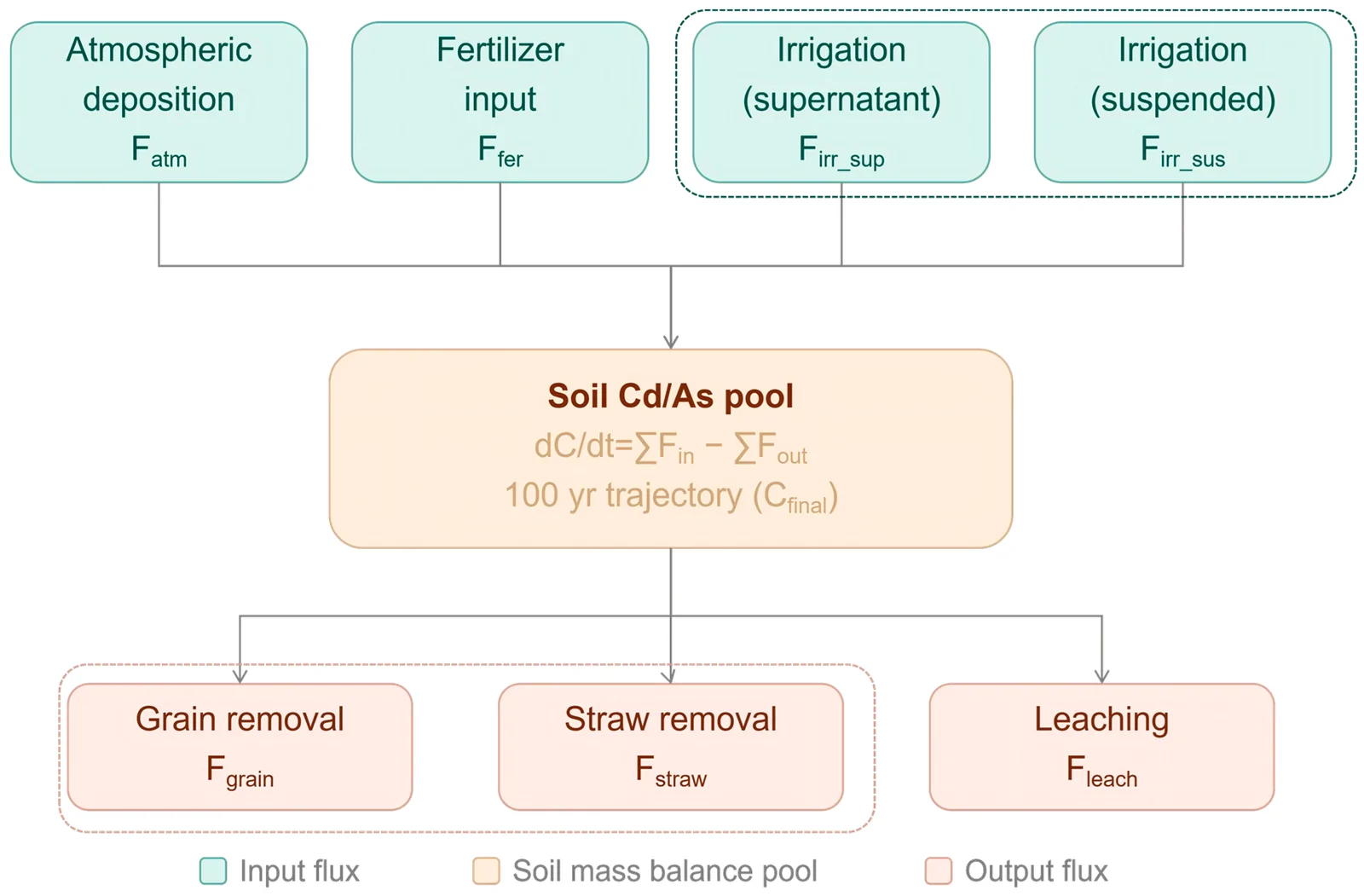
Schematic structure of the refined flux balance model for Cd and As input–output fluxes.

Figure 2 presents a schematic overview of the refined flux balance model. Within this framework, the irrigation input was explicitly split into supernatant and suspended solids, while crop removal was partitioned into grain and straw. These input and output pathways are integrated into the central soil Cd/As mass balance to generate the 100-year concentration projection. The calculation formulas for different pathways in the model are detailed below.

(1) Atmosphere

(1) $$F_{atm}=\frac{C_{q}\times M\times 365}{30\times 100}$$

where *F_atm_* is the flux of atmospheric deposition (g·ha^−1^·yr^−1^); *C_q_* is the content of PTEs in dustfall (mg·kg^−1^) (Table S4); *M* is the amount of dustfall (t·km^−2^, (30 d)^−1^) (Table S4).

(2) Irrigation

(2) $$F_{irr\_sup}=\sum C_{irr\_sup}\times W_{irr}\times \eta \times 10^{4}$$

(3) $$F_{irr\_sus}=\sum C_{irr\_sus}\times S_{sus}\;{\times W}_{irr}\times \eta \times 10^{-2}$$

where *F_irr_sup_* and *F_irr_sus_* are the fluxes of PTEs from supernatant and suspended solids in irrigation, respectively (g·ha^−1^·yr^−1^); *C_irr_sup_* is the content of PTEs in the supernatant of irrigation (mg·L^−1^) (Table S5); *C_irr_sus_* is the content of PTEs in the suspended solids in irrigation (mg·kg^−1^) (Table S5); *S_sus_* is the content of suspended solids in irrigation (mg·L^−1^); *W_irr_* is the usage of irrigation each year (m^3^·m^−2^); *η* is the irrigation water utilization coefficient. *W_irr_* and *η* are from Ningxia Water Resources Bulletin (2021) [26] (Table S6). Because measured suspended-solid PTEs concentrations were unavailable in sufficient quantities, the PTE concentrations in sediment were used as a proxy for those in suspended solids.

(3) Fertilizer

(4) $$F_{Fer}=\sum C_{Fer}\times W_{Fer}\times 10^{-3}$$

where *F_Fer_* is the flux of PTEs in fertilizers (g·ha^−1^·yr^−1^); *C_Fer_* is the content of PTEs in each fertilizer (mg·kg^−1^) (Table S7); *W_Fer_* is the usage of each fertilizer (kg·ha^−1^·yr^−1^) (Table S8).

(4) Crop harvesting

(5) $$F_{grain}=\sum C_{grain}\times Y_{grain}\times 10^{-3}$$

(6) $$F_{straw}=\sum C_{straw}\times \frac{Y_{grain}}{\varepsilon }\times 10^{-3}$$

where *F_grain_* and *F_straw_* are the fluxes of PTEs from grain and straw harvesting, respectively (g·ha^−1^·yr^−1^); *C_grain_* and *C_straw_* are the contents of PTEs in grain and straw, respectively (mg·kg^−1^) (Table S9); *Y_grain_* is the yield of crop (kg·ha^−1^·yr^−1^), from the Ningxia Statistical Yearbook [27] (Table S10); ε is the ratio of grain to straw of crops [28] (Table S11).

(5) Leaching

Following Mi et al. [29], the flux of PTEs from leaching was calculated using Equation (7):

(7) $$F_{lea}=10\times (P+W_{irr}\times (1-\eta )-E)\times C_{w})$$

where *F_lea_* is the flux of PTEs from leaching (g·ha^−1^·yr^−1^); *P* is the total annual precipitation (mm·yr^−1^); W*_irr_* is the total annual irrigation (mm·yr^−1^); *η* is the irrigation water utilization coefficient; *E* is the total annual evapotranspiration (mm·yr^−1^); *C_W_* is the metal concentration in the soil solution (mg·L^−1^) (Table S12). The annual precipitation and the annual evapotranspiration were obtained from the Ningxia Meteorological Bureau (Table S13).

(6) The net flux of PTEs in soil

(8) $$△F_{soil}=\sum F_{in}-\sum F_{out}$$

where *∆F_soil_* is the net flux of PTEs in soil (g·ha^−1^·yr^−1^); *F_in_* is the input flux of PTEs in soil (g·ha^−1^·yr^−1^); *F_out_* is the output flux of PTEs in soil (g·ha^−1^·yr^−1^).

(7) The prediction of PTEs content based on flux balance model

(9) $${HM}_{soil,i}={HM}_{soil,i-1}+\frac{△F_{soil}}{W_{soil}}$$

where ${HM}_{soil,i}$ and ${HM}_{soil,i-1}$ are the contents of PTEs in soil in the i-th and i-1-th year, respectively (mg·kg^−1^); ∆*F_soil_* is the net flux of PTEs in soil (g·ha^−1^·yr^−1^); *W_soil_* is the weight of soil (t·ha^−1^). *W_soil_* is 2300 t·ha^−1^. It is assumed that the input of exogenous PTEs into the farmland soil accumulates only at a depth of 20 cm, and the soil bulk density is 1.15 t·m^−3^.

Descriptive statistics of As and Cd concentrations and fluxes in various media are presented in Table S14.

### 2.5. Health Risk Assessment Based on Soil Exposure

The health risks of Cd and As were assessed using the health risk assessment model for soil exposure developed by the USEPA [30]. The local populations were grouped into children and adults due to their different physiological and behavioral characteristics. The average daily intake (ADI) of Cd and As via three exposure pathways (ingestion, dermal contact, and inhalation), in mg·kg^−1^·d^−1^, was calculated using the following equations:

(10) $${ADI}_{ing}=\frac{C\times {IR}_{ing}\times EF\times ED}{BW\times AT}\times 10^{-6}$$

(11) $${ADI}_{derm}=\frac{C\times SA\times AF\times ABF\times EF\times ED}{BW\times AT}\times 10^{-6}$$

(12) $${ADI}_{inh}=\frac{C\times {IR}_{inh}\times EF\times ED}{PEF\times BW\times AT}$$

where *C* is the concentration of As or Cd in the soil (mg·kg^−1^), *IR_ing_* represents the soil ingestion rate (mg·day^−1^); *EF* is the exposure frequency (day·yr^−1^); *ED* is the exposure duration (yr); *BW* denotes the average body weight (kg); *AT* is the average time (d); *SA* is the exposed skin area (cm^2^); *AF* is the skin adherence factor (mg·cm^−2^); *ABF* is the dermal adsorption factor; *IR_inh_* signifies the inhalation rate (m^3^·day^−1^); *PEF* is the particle emission factor (m^3^·kg^−1^). The values of these exposure-related parameters are provided in Table S15. It should be noted that AT takes different values depending on whether ADI is used to calculate the hazard index (using AT_HI_) or the total carcinogenic risk (using AT_CR_), as specified in Table S15.

The total carcinogenic and non-carcinogenic risks through the combined three exposure pathways are characterized as total carcinogenic risk (TCR) and hazard index (HI), respectively, which can be calculated using Equations (13) and (14):

(13) $$TCR=\sum CR=\sum {ADI}_{ij}\times {SF}_{ij}$$

(14) $$HI=\sum HQ=\sum \frac{{ADI}_{ij}}{{RfD}_{ij}}$$

where the carcinogenic risk (CR) and hazard quotient (HQ) represent the carcinogenic and non-carcinogenic risks of individual exposure pathways, respectively, and *ADI_ij_, RfD_ij_*, and *SF_ij_* denote the average daily intake, the chronic reference dose, and slope factor for each metal via each exposure pathway, respectively. The specific values of these parameters are provided in Table S16. For HQ or HI, a value greater than 1 indicates unacceptable risk. For CR or TCR, a value below 1 × 10^−6^ is considered negligible risk, values between 1.0 × 10^−6^ and 1.0 × 10^−4^ indicate notable risk, and values exceeding 1.0 × 10^−4^ are deemed unacceptable [31,32].

### 2.6. Sensitivity and Uncertainty Analysis

To systematically quantify the propagation of parameter uncertainties and identify the dominant drivers governing the 100-year projected PTE concentrations (C_final_) in regional soils, a sensitivity analysis (SA) was conducted. Utilizing a Monte Carlo simulation framework with 10,000 iterations, all primary model inputs were probabilistically characterized using normal distributions based on regional field monitoring datasets.

The parameterization rules and boundary conditions for model inputs across the four regions (DB, HGZ, SK, and TG) were established as follows. Input/Output Fluxes: Site-specific annual fluxes, including atmospheric deposition, irrigation supernatant, suspended solids, fertilizer inputs, crop removal, and leaching, were characterized by normal distributions fitted to regional monitoring data (mean ± SD). For parameters lacking an empirical SD, a prescriptive coefficient of variation (CV) of 10% was assigned to describe their potential operational fluctuations. To ensure physical plausibility, a truncation constraint (max (0, X)) was applied to exclude stochastically generated negative values.

The baseline soil mass of the 20 cm plow layer was prescribed with a mean of 2300 t·ha^−1^ and a CV of 5%, reflecting localized bulk density heterogeneity.

The sensitivity of C_final_ for Cd and As to individual parameters was quantified using Pearson correlation coefficients (*r*). A positive r indicates that increasing the parameter exacerbates soil PTE accumulation, while a negative r indicates enhanced removal. Parameters with |*r*| ≥ 0.10 were classified as primary or secondary control factors and considered significant contributors to predictive uncertainty; parameters with |*r*| < 0.10 were deemed negligible given their marginal influence on long-term projection variability.

### 2.7. Data Analysis and Visualization

Kruskal–Wallis tests were used to evaluate regional differences among the four typical irrigation areas, followed by Dunn–Holm post hoc pairwise comparisons when the overall test was significant. All data processing and flux-balance calculations were executed in Python 3.12.3 utilizing pandas 2.3.1, numpy 1.26.4, and scipy 1.14.1. Statistical plots were rendered using seaborn 0.13.2 and Matplotlib 3.11.0.

## 3. Results and Discussion

### 3.1. The Concentrations of Cd and As in Soils

Soil properties were measured and listed in Table S17. The surface soils were alkaline, with pH ranging from 8.06 to 8.79. Organic matter (OM) ranged from 12.60 to 23.50 g·kg^−1^, with an average of 16.64 g·kg^−1^, and cation exchange capacity (CEC) ranged from 6.20 to 12.20 cmol (+)·kg^−1^, with an average of 9.52 cmol (+)·kg^−1^.

As shown in Table 1, the concentration of As in soils was 7.42~13.50 mg·kg^−1^, which is lower than the screening value of the Soil Environmental Quality Risk Control Standard for Soil Contamination of Agricultural Land (GB 15618-2018) (20 mg·kg^−1^) [20]. The highest concentration was observed in HGZ (10.69 ± 1.05 mg·kg^−1^), while the lowest was found in DB (9.66 ± 1.79 mg·kg^−1^). The Nemerow index (PI) of As in soil was 0.62~1.13. The average PI values of As in different areas decreased in the following order: HGZ (0.90) > TG (0.86) ≈ SK (0.85) > DB (0.81); however, these regional differences were not statistically significant. The concentration of Cd in soils was 0.07~0.24 mg·kg^−1^ (Table 1), which is also lower than the screening value specified in GB15618-2018 (0.30 mg·kg^−1^). The highest concentration was observed in SK (0.18 ± 0.06 mg·kg^−1^), while the lowest was found in TG (0.11 ± 0.03 mg·kg^−1^). The PI of Cd in soil was 0.63~2.14. The order of average Cd PI values in different areas was as follows: SK (1.56) > DB (1.16) > TG (1.02) ≈ HGZ (1.03); however, these regional differences were not statistically significant. Overall, As and Cd concentrations in the study soils did not exceed the agricultural land risk screening values. The PIs of As and Cd at some sampling points were higher than 1, indicating a cumulative effect of these metals in the soils. Notably, the PI values of Cd were higher than those of As, showing that human activities have a more significant impact on soil Cd than on As, consistent with the results reported by Chen et al. [19]. The pollution load index (PLI) of only the SK area was greater than 1 (1.17), which was mainly attributed to the relatively high enrichment of cadmium (Cd) in this region, while the PLI values of the other three areas were all below 1. The geoaccumulation indices (I_geo_) of Cd and As in soil samples from all four regions were lower than 1, indicating that soils were classified as ranging from uncontaminated to negligibly enriched [33]. In summary, while the four representative study areas currently remain below regulatory screening values, they exhibit a distinct signature of historical accumulation.

### 3.2. Inventory of Cd and As Inputs

(1) Atmospheric deposition

The As flux of atmospheric deposition was 13.10~102.71 g·ha^−1^·yr^−1^, with an average of 30.5 g·ha^−1^·yr^−1^ (Table S18), larger than the average in China (28 g·ha^−1^·yr^−1^) [34], but still far less than that in Hunan Province (99.5 g·ha^−1^·yr^−1^) [6]. The order of the input As fluxes from atmospheric deposition in the four typical regions is SK > HGZ > DB > TG (Table 2). The Cd flux of atmospheric deposition was 0.2~1.86 g·ha^−1^·yr^−1^, with an average of 0.67 g·ha^−1^·yr^−1^ (Table S18), which is lower than the national average (4.0 g·ha^−1^·yr^−1^) reported by Luo et al. [34] and less than that observed in Hunan Province (16.65 g·ha^−1^·yr^−1^) [6]. The input Cd fluxes via atmospheric deposition across the four study areas followed a descending order of SK > DB > HGZ > TG (Table 2), with the flux in SK being significantly higher than that in TG (*p* = 0.011). The heightened fluxes of Cd and As in SK are likely attributed to its substantial dustfall (12.46 t·km^−2^·(30 d)^−1^) combined with high content of PTEs in the deposited dust (Table S4). Intensive fossil fuel combustion and nonferrous metal smelting represent the predominant drivers of Cd and As emissions in the SK and DB regions, owing to their heavy industrial infrastructure [35]. Similarly, Miletić et al. [36] identified atmospheric emissions from the oil-refining industry as the dominant source of soil As, Cd, Hg, and Cr. However, given the relatively short sampling duration, the estimated annual atmospheric deposition flux derived from this period may not fully capture seasonal variability, introducing a potential underestimation that warrants cautious interpretation.

(2) Irrigation

As found by Hou et al. [1], irrigation, in addition to mining and smelting, is also a significant factor contributing to the exceedance of heavy metal concentrations in soil. The traditional mass balance model often treats irrigation water as an undifferentiated bulk phase. This approach cannot distinguish the contributions of supernatant-associated and suspended-solid-associated Cd and As, especially in high-sediment river irrigation systems (such as the Yellow River irrigation area in Ningxia). To overcome this constraint and more accurately quantify the total PTE input flux of irrigation water, the irrigation flux was partitioned into supernatant and suspended solids in irrigation water in the refined flux calculation model.

The input flux from supernatant Cd and As was 0.21~2.66 g·ha^−1^·yr^−1^ (average: 0.48 g·ha^−1^·yr^−1^; median: 0.40 g·ha^−1^·yr^−1^) and 7.44~49.35 g·ha^−1^·yr^−1^ (average: 15.22 g·ha^−1^·yr^−1^; median: 9.55 g·ha^−1^·yr^−1^) (Table S19), exceeding their respective national average of 0.20 g·ha^−1^·yr^−1^ and 1.80 g·ha^−1^·yr^−1^ [34]. For the suspended solids phase, the input flux was 0.05~6.63 g·ha^−1^·yr^−1^ for Cd (average: 0.68 g·ha^−1^·yr^−1^; median: 0.39 g·ha^−1^·yr^−1^) and 3.52~391.17 g·ha^−1^·yr^−1^ for As (average: 39.44 g·ha^−1^·yr^−1^; median: 20.12 g·ha^−1^·yr^−1^) (Table S19). A paired Wilcoxon signed-rank test showed that the flux of As via suspended solids was significantly higher than that via the supernatant of irrigation water (*p* = 0.003), whereas no statistically significant difference was detected between the Cd flux via suspended solids and that via the supernatant (*p* = 0.95). As highlighted by Liu et al. [37], particulate suspended matter serves as a key carrier of PTEs in water bodies. The concentrations of suspended solids in irrigation water were 70~11,134 mg·L^−1^ (Table S5), with 87.0% to 89.5% of sites exceeding the limits (100 mg·L^−1^) set by the Standard for Farmland Irrigation Water Quality (GB 5084-2021) [38]. Meanwhile, the concentrations of As and Cd in the supernatant were lower than the limits of As (0.1 mg·L^−1^) and Cd (0.01 mg·L^−1^), while the average concentrations of As and Cd in the sediment were 7.75 and 0.13 mg·kg^−1^, respectively. These findings align with Xie et al. [39], who found that PTE contents in sediment of the Yellow River remained high since 2013 and showed little change over time. The high concentrations of suspended solids and associated PTEs led to elevated fluxes from suspended solids.

(3) Fertilizer

Phosphate and compound fertilizers were exclusively considered as the fertilizer inputs in this study, owing to their elevated PTEs concentrations relative to other fertilizer types [40]. The analytical results (Table S7) further revealed that higher heavy metal concentrations were present in both phosphorus-containing fertilizers and compound fertilizers. Although the contents of Cd and As in the fertilizer comply with national standards (Table S7), the total fluxes from fertilizers for Cd and As were 0.36~0.45 g·ha^−1^·yr^−1^ and 13.76~19.01 g·ha^−1^·yr^−1^ (Table S20), respectively. The average Cd flux (0.39 g·ha^−1^·yr^−1^) from fertilizers is comparable to that reported for Hainan Island, while the average As flux (16.09 g·ha^−1^·yr^−1^) exceeds the levels observed there [41]. Notably, the highest PTEs fluxes from fertilizers were recorded in DB, whereas the lowest fluxes were recorded in TG (Table 2). This spatial heterogeneity is directly driven by the higher application rates of phosphate and compound fertilizers in DB (Table S8), which may be related to the agricultural production characteristics of each township [42].

(4) Relative contributions of the Cd and As fluxes input pathways

**Figure 3:**
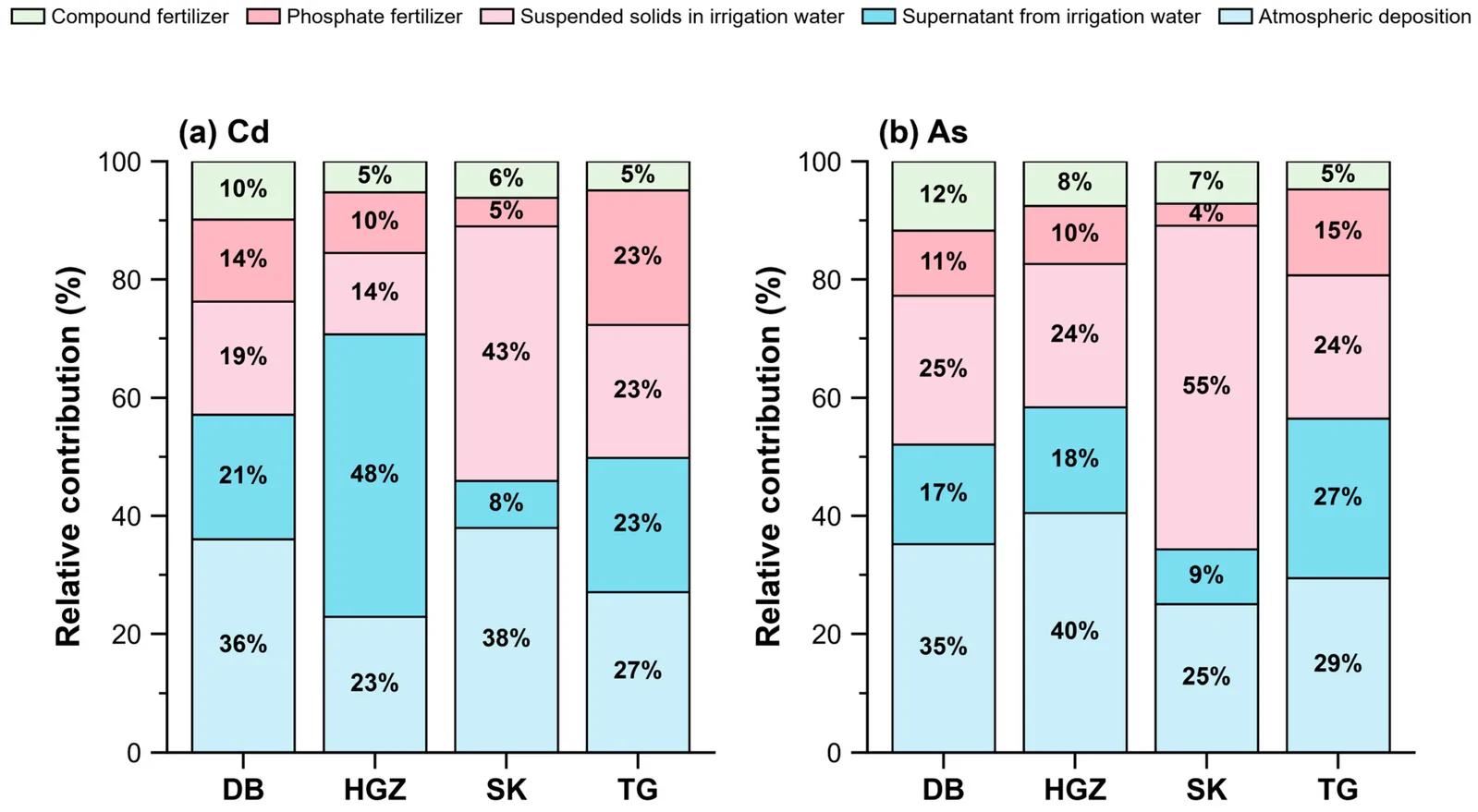
Relative contributions of Cd (**a**) and As (**b**) input pathways.

The average total input flux of Cd in the four typical regions was 1.31~3.25 g·ha^−1^·yr^−1^, lower than the national average (11.61 g·ha^−1^·yr^−1^) [34]. The average total input flux of As in the four typical regions was 71.43~146.99 g·ha^−1^·yr^−1^, higher than the national average (48.57 g·ha^−1^·yr^−1^) [34]. As Figure 3 shows, the main input pathway of Cd is irrigation and atmospheric deposition. The irrigation input fluxes accounted for 40~62%, especially the suspended solids in irrigation. The Cd content in the sediment of the lower reaches of the Yellow River in Ningxia exceeded its background value [43]. In addition, the atmospheric deposition and fertilizer input fluxes accounted for 23~38% and 11~28%, respectively. The relative contribution of atmospheric deposition is lower than the average relative contribution of atmospheric in China (70%) [14] and Europe (68%) [44], especially in Hunan province. As Fan et al. [11] proposed, economically developed regions with intensive industrial and mining activities tend to exhibit high Cd flux through atmospheric deposition. As Figure 3 shows, the main input pathway of As is irrigation and atmospheric deposition. The irrigation input fluxes accounted for 42~64%. In addition, the atmospheric deposition and fertilizer input fluxes accounted for 25~40% and 11~23%, respectively. In general, irrigation and atmospheric deposition are the main sources of potentially toxic elements fluxes in soil. Similarly, the combined application of the PMF model and multivariate statistical analysis identified that irrigation water and sediment from the Yellow River exhibited the highest contribution rate to potentially toxic elements (42.25%), followed by industrial and transportation activities (16.06%) [45].

### 3.3. Inventory of Cd and As Outputs

(1) Crop Harvesting

In the four typical regions, the Cd flux from grain and straw was 0.004~1.04 g·ha^−1^·yr^−1^ and 0.01~1.39 g·ha^−1^·yr^−1^ (Table S21), with regional average values of 0.16 g·ha^−1^·yr^−1^ and 0.60 g·ha^−1^·yr^−1^, respectively. In the Ningxia region, where maize cultivation is predominant, the cadmium (Cd) output flux in maize grains is lower than that in wheat grains (0.21 g·ha^−1^·yr^−1^, range: 0.12~0.52 g·ha^−1^·yr^−1^) and rice grains (0.45 g·ha^−1^·yr^−1^, range: 0.05~1.25 g·ha^−1^·yr^−1^) from the Yangtze River Delta region [15]. The As flux from grain and straw was 0.07~4.12 g·ha^−1^·yr^−1^ and 1.04~40.16 g·ha^−1^·yr^−1^ (Table S21), and the regional average was 0.71 g·ha^−1^·yr^−1^ and 11.14 g·ha^−1^·yr^−1^, respectively. The highest Cd and As fluxes from grain and straw harvesting were observed in TG, whereas the lowest were recorded in HGZ (Table 3). Generally, the flux of PTEs from straw harvesting was significantly higher than that from grain (*p* = 7.63 × 10^−6^). The notably higher concentrations of PTEs in straw compared to grain for these four types of crops, coupled with the greater yield of straw relative to grain [46], lead to substantially higher PTE fluxes from straw than from grain. This stems from variations in crop types and soil characteristics across different regions, which lead to differences in crop yields and PTE concentrations [47].

(2) Leaching

The Cd output flux of leaching is 0.11~1.43 g·ha^−1^·yr^−1^, with an average value of 0.35 g·ha^−1^·yr^−1^ (Table S22). The As flux of leaching is 3.45~32.05 g·ha^−1^·yr^−1^, with an average value of 8.60 g·ha^−1^·yr^−1^ (Table S22). These results are comparable to the leaching flux of As (4.0~40.5 g·ha^−1^·yr^−1^) in Heilongjiang Province, while lower than that of Cd (41.5~872.6 g·ha^−1^·yr^−1^) [48]. It has also been shown that the leaching of certain PTEs is largely pH-dependent, with lower mobility under slightly alkaline conditions [49]. The order of average Cd flux output of leaching in four typical regions is HGZ > SK > TG ≈ DB (Table 3). The order of average As flux output of leaching in four typical regions is TG > HGZ > SK > DB (Table 3).

(3) Relative contributions of the Cd and As flux output pathways

**Figure 4:**
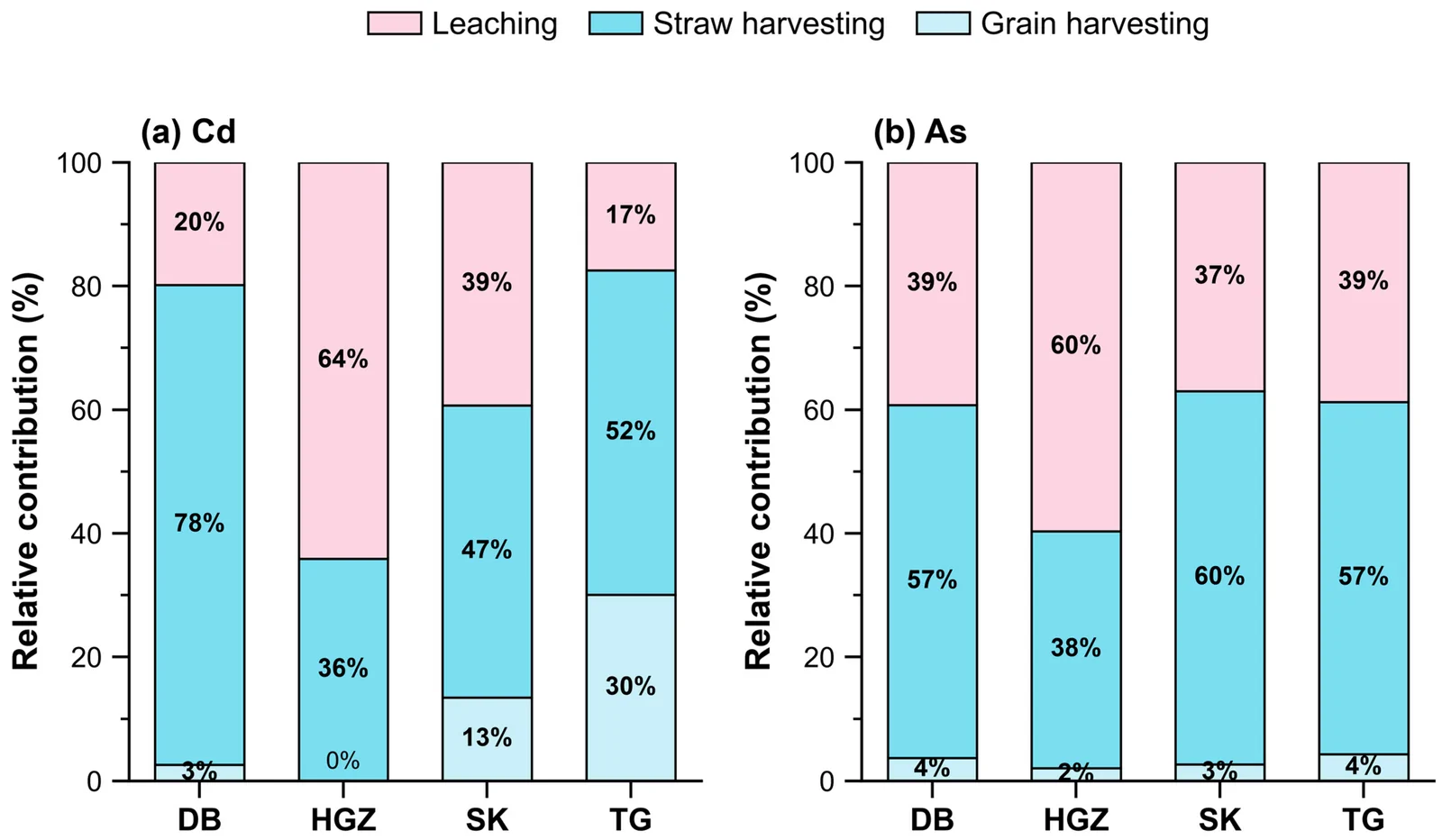
Relative contributions of Cd (**a**) and As (**b**) output pathways.

Two straw management scenarios were considered in this study: straw return, in which only grain is harvested and straw remains in the field, and straw off-field, in which both grain and straw are removed. The average total output flux of Cd and As in the four typical areas was 0.89~1.43 g·ha^−1^·yr^−1^ and 13.81~33.86 g·ha^−1^·yr^−1^, respectively, with straw off-field. The average total output flux of Cd and As in the four typical areas was 0.26~0.68 g·ha^−1^·yr^−1^ and 5.93~14.59 g·ha^−1^·yr^−1^, respectively, with straw return. As shown in Figure 4, crop harvest is the main pathway for the output of cadmium from the soil, accounting for 36~80% for Cd and 40~63% for As. In particular, the proportion of removal through straw harvest is much higher than that through grains. This is consistent with previous studies, which showed that the removal of Cd through rice straw was several times greater than that through grains [6,14]. In HGZ, leaching accounted for 64% and 60% of the Cd and As output fluxes, respectively, while crop harvesting (including both grain and straw removal) accounted for only 36% and 40% of the Cd and As output fluxes, respectively. HGZ is located in Shizuishan, which is a well-known coal mining area. Previous studies showed that PTEs in the coal mine after mining gradually transformed from an inert state to an active state as the weathering process occurred [50].

### 3.4. Prediction of Cd and As in Soil Based on Flux Balance

The average net Cd flux was −0.11~2.36 g·ha^−1^·yr^−1^ and 0.64~2.79 g·ha^−1^·yr^−1^ under straw off-field and straw return (Table 4), respectively. The average net Cd flux in TG is negative, indicating that the concentration of Cd in soil decreased gradually. On the contrary, the average net flux in other areas was positive, indicating the PTE concentrations increased gradually. Notably, the highest average net flux in SK showed that the accumulation rate of Cd in soil was higher than that in other areas. The average net As flux was 37.57~131.53 g·ha^−1^·yr^−1^ and 56.84~140.86 g·ha^−1^·yr^−1^ under straw off-field and straw return (Table 4), respectively. As showed an accumulation tendency in soil, and the accumulation rate followed the order: SK > HGZ > DB > TG. In general, the accumulation rate of PTEs in soil could be decreased under straw off-field, and even the concentrations of PTEs in soil could be reduced. The highest accumulation rate of Cd and As in soil is in SK and HGZ. This discrepancy may be partly associated with differences in local industrial activities, as industrial enterprises in SK are primarily nonferrous metal smelting enterprises, whereas those in HGZ are concentrated in ferroalloy smelting enterprises. Previous studies have suggested that the sources of As and Cd in the soil around coal chemical enterprises are mainly from the emissions of flue gas from these enterprises. Similarly, Zhang et al. [9] used scenario-based random forest modeling to quantify that non-ferrous mining (68.0%), energy consumption (15.8%), and smelting (13.2%) collectively drive over 97% of China’s rising soil arsenic. This is because Cd contained in coal combustion is easily volatilized, while As is transformed into gaseous oxides and exists in fly ash [51]. According to Li et al. [52], soil near lead–zinc smelters, which are non-ferrous metal smelters, exhibited the most severe pollution levels of Cd and As. Industrial activities such as smelting of steel and non-ferrous metals are important sources of anthropogenic potentially toxic elements [53].

**Figure 5:**
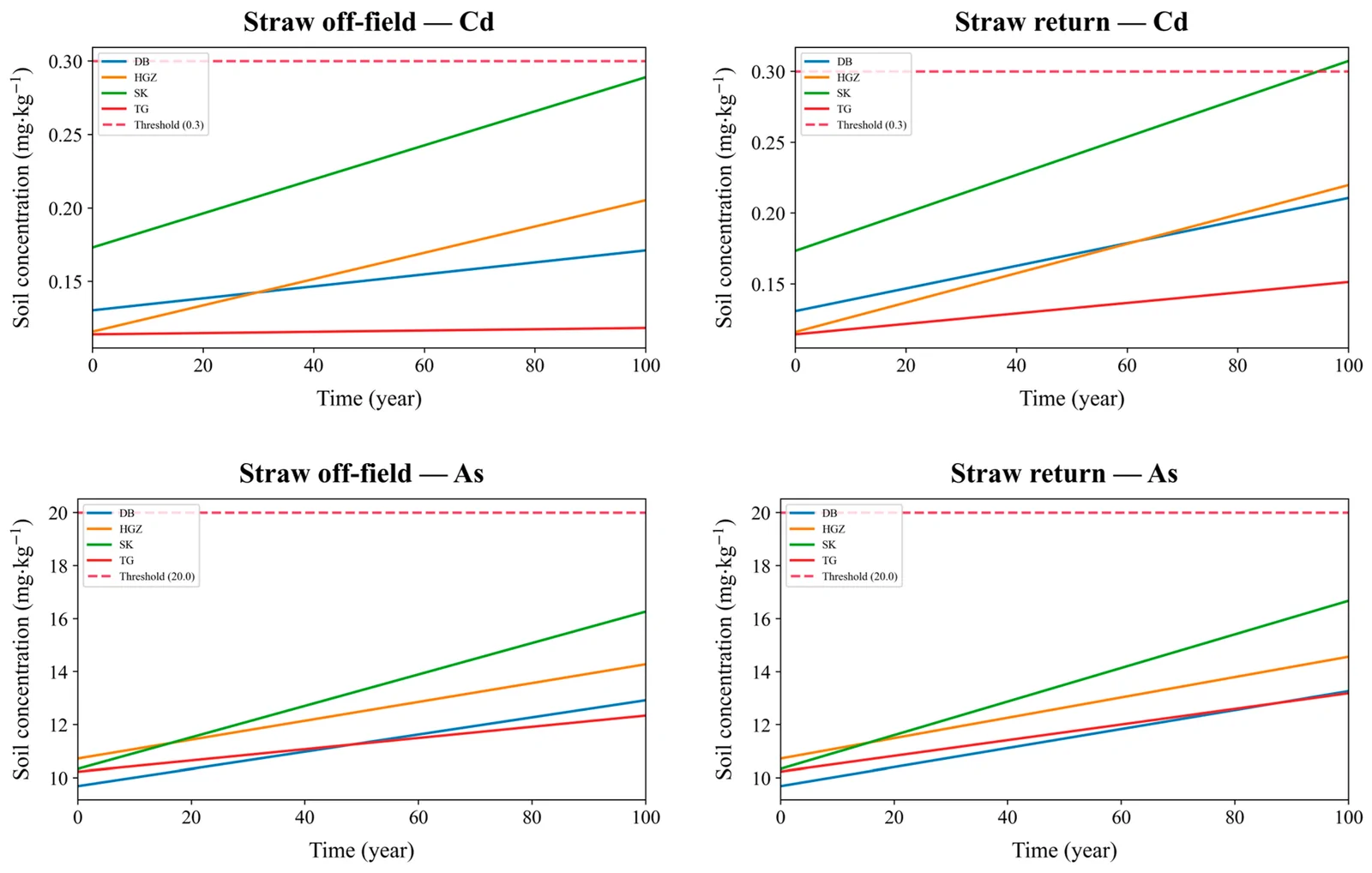
Projected Cd and As concentrations in soil under different scenarios. Lines represent projections using mean annual input and output fluxes measured during the 2023 monitoring period. Dashed horizontal lines represent GB 15618-2018 risk screening values (Cd: 0.30 mg·kg^−1^ for soil pH ≤ 7.5; As: 20 mg·kg^−1^ for paddy fields).

Under the current environmental and management policies, the concentrations of Cd and As predicted based on the current net flux after 100 years did not exceed the corresponding screening value of the Soil Environmental Quality Risk Control Standard for Soil Contamination of Agricultural Land (GB 15618-2018) [20] (Figure 5). However, the concentrations of Cd and As in SK soil are close to the screening value. As Figure 5 shows, under straw return, the accumulation rate of Cd and As in soil is faster, and with the extension of the accumulation time, the impact of straw return becomes more pronounced, consistent with Wei et al. [54]. In particular, the growth rate of Cd and As in SK is also higher than that in the other three regions. At present, the Cd and As concentrations in the soil of TG are low, and the accumulation rate is low. The input and output fluxes in the four regions showed great spatial heterogeneity, which led to great differences in the accumulation rate of Cd and As in soil under different scenarios.

To validate the model’s temporal reliability, the back-calculation of the historical accumulation periods from regional baseline levels to current ambient concentrations was conducted and is displayed in Table S23. The back-calculated Cd accumulation periods for SK and DB (55~66 years) align seamlessly with the local history of intensified non-ferrous smelting and coal mining since the 1960s–1970s, while the positive As net fluxes successfully reconcile current below-background concentrations with the forward-simulated early net-accumulation phase. The resulting timelines exhibit robust environmental plausibility.

### 3.5. Sensitivity Analysis of Flux Balance Model

**Figure 6:**
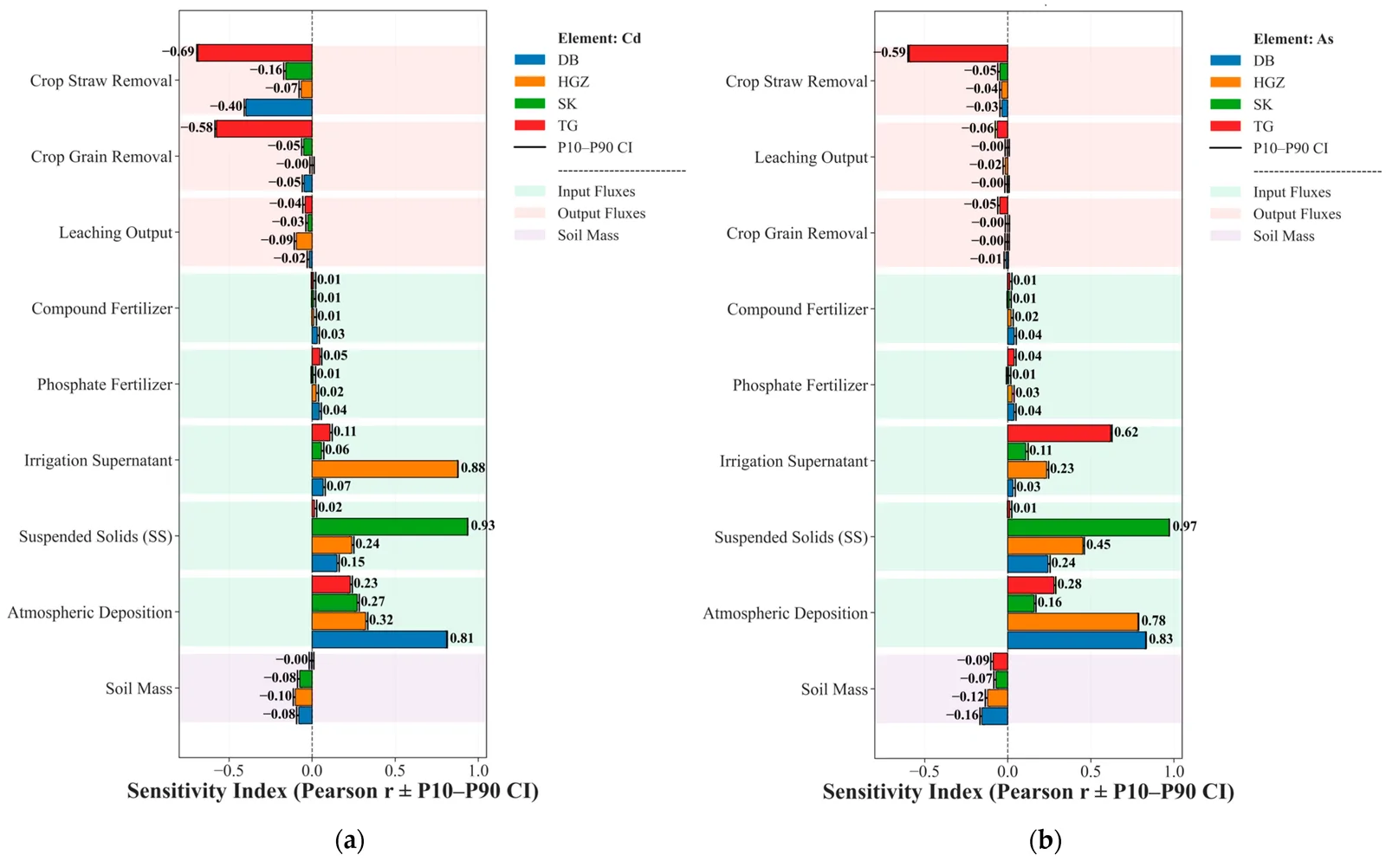
Sensitivity analysis of the Cd (**a**) and As (**b**) flux balance models. Bars: Pearson r (median of 10,000 Monte Carlo iterations). Black lines with caps: P10–P90 bootstrap confidence intervals.

The sensitivity analysis (SA) revealed pronounced spatial heterogeneity in the dominant drivers of long-term Cd and As accumulation across the four sub-regions. Atmospheric deposition (F*_atm_*) emerged as a consistently critical input driver, particularly in DB and HGZ, where it exhibited high positive correlation coefficients (*r* up to 0.83 for Cd and 0.81 for As) (Figure 6). In HGZ, this pronounced sensitivity is consistent with the immediate proximity of the sampled farmland to a concentrated industrial zone (Figure 6b). This spatial pattern, together with Ningxia’s broader positioning within an intermediate stage of industrialization dominated by resource-, energy-, and heavy chemical-based industries, indicates that industrial emission control represents the most effective long-term management strategy for these areas.

Crucially, by segregating irrigation water into fluid and solid phases, the refined model captured highly divergent regional pathways that conventional bulk-water models typically mask. In SK, suspended solids (F*_irr_sus_*) dominated overwhelmingly (*r* = 0.94 for Cd, *r* = 0.97 for As), whereas irrigation supernatant (F*_irr_sup_*) was negligible, demonstrating that suspended-solid deposition was the dominant PTEs input pathway. Conversely, in HGZ, irrigation supernatant was the primary driver for Cd accumulation (*r* = 0.88), highlighting dissolved-phase contamination. These contrasting results support the methodological necessity of phase-separated irrigation modeling to guide targeted regional remediation.

On the output side, crop straw removal (F*_straw_*) introduced a major, region-specific negative feedback loop that was most pronounced in TG (*r* = −0.78 for Cd, *r* = −0.71 for As). This substantial negative correlation indicates that the long-term risk profile in TG is highly sensitive to post-harvest residue management: transitioning to full straw incorporation would eliminate this primary removal pathway and accelerate soil PTE accumulation.

In contrast, other mass balance components, including fertilizer inputs (F*_fer_*) and leaching output (F*_lea_*), consistently maintained minor sensitivity indices (|r| < 0.10) across all study locations. These results suggest that despite their baseline contributions, seasonal or empirical fluctuations of these minor parameters exert negligible influence on the threshold-exceedance risk over a 100-year projection horizon.

These location-specific sensitivities enable the formulation of spatially targeted environmental strategies, including sediment management, source-water substitution, emission controls, and residue retention, tailored to each sub-region.

### 3.6. The Assessment of Health Risk of Cd and As via Soil Exposure

**Figure 7:**
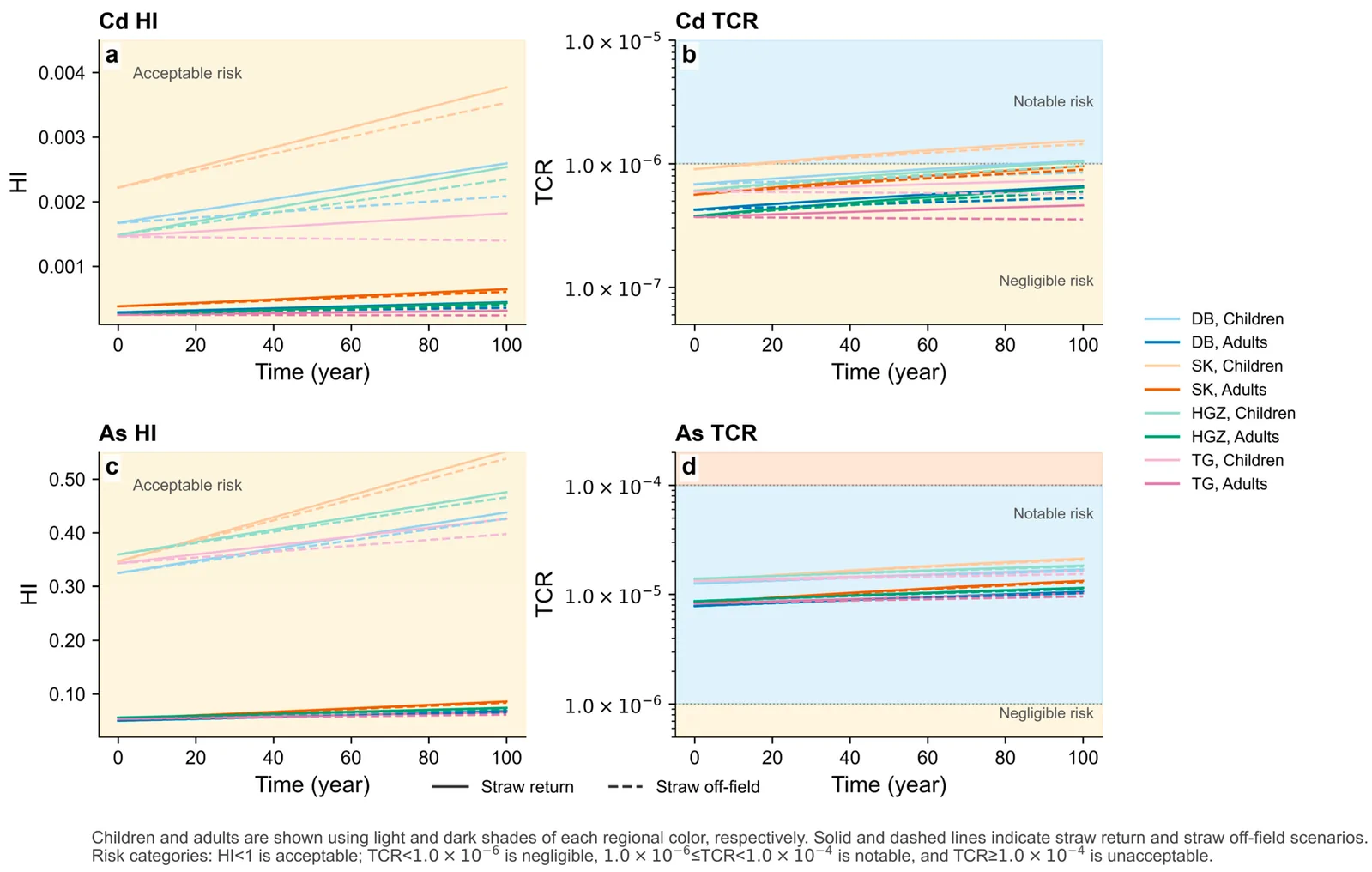
The prediction of HI and TCR of Cd and As in soil under different scenarios. Colors indicate study areas, line types indicate straw management scenarios, and light/dark shades indicate children/adults. Background colors indicate risk categories.

The assessment results indicated that the hazard indices (HI) of Cd and As were within acceptable non-carcinogenic risk levels for both children and adults. However, the As-specific carcinogenic risk was already within the notable risk range in the initial year and remained notable throughout the 100-year projection, with children being more vulnerable compared to adults. In contrast, the Cd-specific carcinogenic risk was generally lower but showed an increasing trend over time (Figure 7). Similar findings of relatively high carcinogenic risks for As but low non-carcinogenic risks for Cd have also been reported in other studies, such as the park soils of Shanghai [24] and agricultural soils surrounding a gold smelting enterprise [55]. For As, children showed higher TCR values than adults across all regions and scenarios, but both receptor groups remained within the notable risk category. Comparing the risk among different regions, HGZ showed the highest initial TCR for As. However, SK exhibited the highest growth rate of As-specific CR due to its highest As net flux. For Cd, children were more sensitive than adults, and several exposure scenarios for children crossed the 1.0 × 10^−6^ threshold from negligible to notable risk during the 100-year projection. Specifically, the Cd-specific CR for children exceeded 1.0 × 10^−6^ in SK under straw return and straw off-field after approximately 16 and 18 years, respectively. In DB and HGZ, this threshold was reached after approximately 85 and 93 years, respectively, both under straw return. In the straw management scenarios, straw off-field resulted in a lower risk growth rate and slightly reduced Cd-related risks in TG. These findings highlight the risk-reduction benefits of straw off-field practices. However, their broader environmental and agronomic trade-offs [56], including impacts on soil fertility, carbon sequestration, and cropland sustainability, should be systematically evaluated before region-wide implementation. Special attention should also be paid to the safe resource utilization of heavy metal-laden straw after removal to prevent secondary pollution during its disposal or recycling.

**Figure 8:**
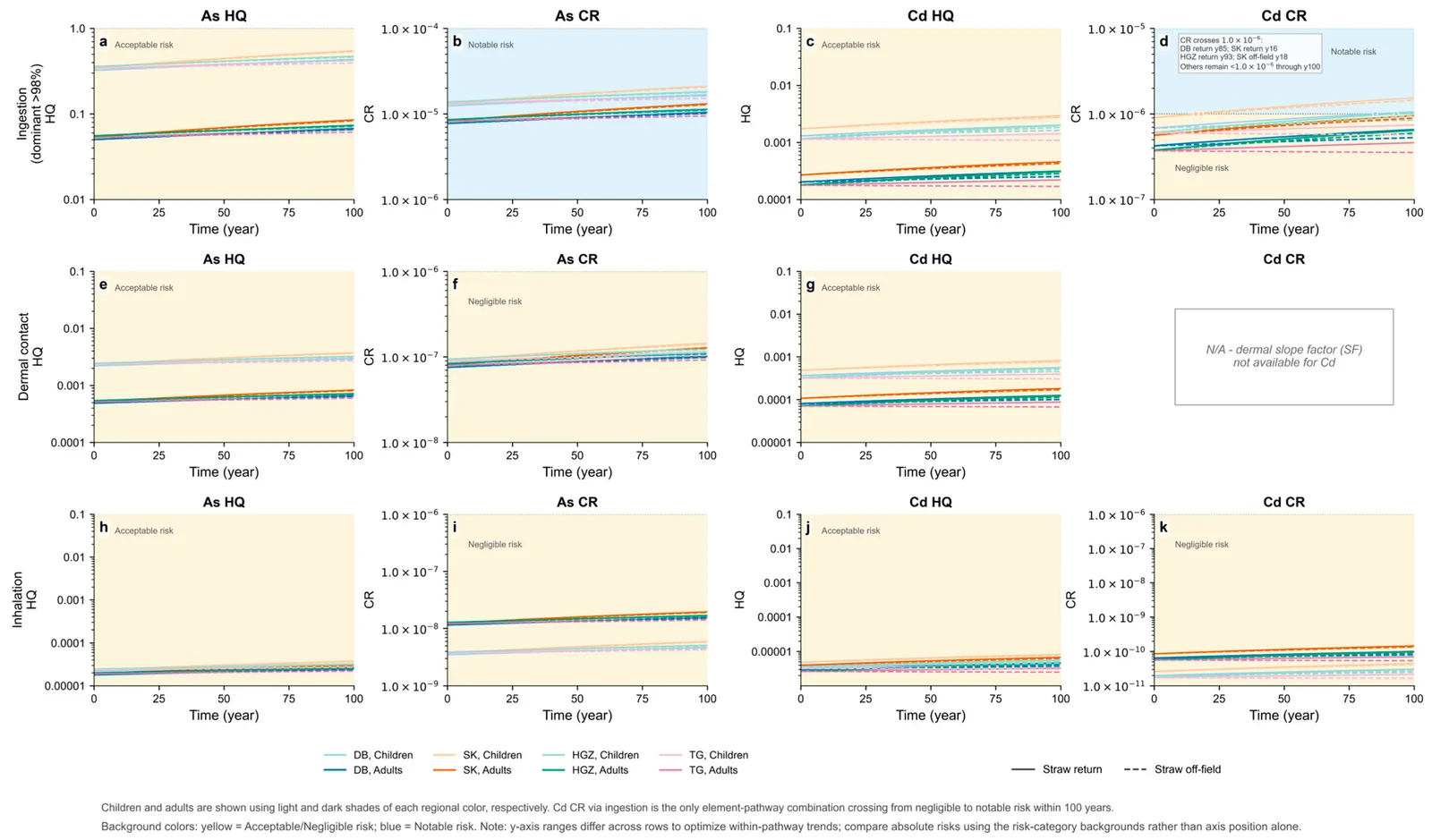
The prediction of HQ and CR of Cd and As in soil via soil ingestion, dermal contact, and inhalation. Colors indicate study areas, line types indicate straw management scenarios, and light/dark shades indicate children/adults. Background colors indicate risk categories.

Health risks were assessed for each exposure pathway (Figure 8). The carcinogenic risk (CR) of As via ingestion was notable for children (ranging from 1.24 × 10^−5^ to 2.11 × 10^−5^) and adults (ranging from 7.73 × 10^−6^ to 1.31 × 10^−5^). In contrast, dermal contact and inhalation generally remained within the negligible risk range. Ingestion accounted for more than 98% of TCR for Cd and As, whereas dermal contact and inhalation contributed only minor proportions (Figures S1 and S2). For HI, ingestion was also the dominant pathway, contributing approximately 98.8~99.2% of the total non-carcinogenic risk, while inhalation was negligible. Notably, dermal contact contributed more substantially to HI for Cd than for As, accounting for approximately 21.8% in children and 28.2% in adults (Figures S1 and S2). This elevated risk in children is likely due to their unique behaviors, such as higher soil ingestion rates and hand-to-mouth activity [57]. Therefore, reducing soil ingestion can effectively reduce soil-exposure risks. As soil Cd and As concentrations increased over time, although they remained below the agricultural soil screening values, the cumulative, multi-pathway, and multi-contaminant nature of human exposure still resulted in notable carcinogenic risk. These results suggest that compliance with agricultural soil screening values, which are designed to protect food-chain safety, does not necessarily guarantee acceptable direct human health risk, particularly for sensitive receptors such as children. Therefore, integrating regulatory screening assessments with quantitative health risk assessment is necessary for comprehensive evaluation in agricultural soil environmental management. Additionally, mitigating PTE inputs through controlling atmospheric deposition and irrigation, as well as adopting straw off-field practices, can effectively limit the increasing concentrations and associated health risks of PTEs.

### 3.7. Limitations and Future Directions

While the sensitivity analysis successfully quantified sample-size-driven uncertainties, several inherent structural and parameter-related limitations warrant consideration for future research.

First, this refined flux balance model assumes static input–output fluxes, fixed crop rotations, and constant irrigation efficiency over the 100-year projection horizon. However, future trajectories could be shifted by long-term environmental and policy dynamics. For instance, climate-driven hydrological extremes might alter Yellow River sediment loads, directly affecting particle-bound inputs in SK, while canal modernization could alter water volumes and phase-associated partitioning. Additionally, potential land-use conversions, agricultural intensification, or mandated straw-return policies would inevitably reshape output fluxes, especially the negative feedback pathways identified in TG. Future work should therefore extend this model by coupling dynamic climate, land-use, and agricultural policy scenario-based projections.

Second, the assumption of homogeneous accumulation within the upper 20 cm plow layer overlooks the influence of soil physicochemical properties on the migration and transformation of PTEs. Mechanisms such as leaching or preferential flow under flood irrigation remain unresolved, which may overestimate near-surface accumulation under high-leaching conditions. Furthermore, both flux modeling and human health risk assessments were parameterized using total metal(loid) concentrations. For risk characterization, using total concentrations instead of bioavailable or bioaccessible fractions represents a conservative, upper-bound estimate of actual hazard, omitting the crucial modulating effects of soil properties. Moreover, since dietary intake via crop consumption was not evaluated, the actual cumulative risk may be underestimated, particularly in regions where local crops constitute a major dietary share. Future studies should integrate bioaccessibility-based assessments with a dietary exposure model that incorporates soil-to-crop transfer factors and site-specific consumption rates.

Additionally, the Monte Carlo analysis was parameterized with truncated normal distributions for all inputs based on reported mean ± SD. However, environmental flux data with high coefficients of variation (e.g., suspended solids flux in SK) are often positively skewed, so this assumption may underestimate upper-tail variability. The limited sample sizes across study areas precluded reliable distributional diagnostics (e.g., Shapiro–Wilk tests, lognormal/gamma fitting), so the current predictions should be interpreted as first-order estimates. Larger multi-year datasets are essential for supporting data-driven distribution selection.

Finally, although the refined model isolated component-specific pathways, it lacks receptor-oriented source apportionment to quantitatively isolate anthropogenic drivers. Integrating quantitative source tracking with speciation-resolved, probabilistic risk assessment frameworks [36] represents a critical future direction to advance from phase-resolved characterization to definitive source-level interventions.

## 4. Conclusions and Implications

This study quantified the fluxes of Cd and As inputs to and outputs from soils in four types of areas currently below agricultural land risk screening values along the Yellow River. Irrigation water (40~64%) and atmospheric deposition (23~40%) were the primary input pathways, while crop harvesting dominated the output fluxes (36~82%). Using a refined flux model, region-specific dominant pathways were identified as atmospheric deposition, suspended solids in irrigation water, irrigation supernatant, and crop straw removal for DB, SK, HGZ, and TG, respectively. Except for Cd in TG under straw off-field, net fluxes of Cd and As were positive across all regions, with the highest accumulation rates in SK and HGZ, where smelting industries are concentrated.

Under current management policies, soil Cd and As concentrations are projected to remain compliant with national standards after 100 years. However, despite acceptable non-carcinogenic risks, the projected combined TCR values remained within the notable carcinogenic risk range, especially for children. Soil ingestion was the dominant exposure pathway, and the Cd-specific CR for children crossed the 1.0 × 10^−6^ threshold from negligible to notable in several scenarios, indicating that compliance with soil quality standards does not necessarily preclude long-term direct human health concerns.

Regional industrial and agricultural structures significantly influenced both the accumulation rates of PTEs and their health risks via soil exposure. To effectively control soil PTE accumulation and associated risks, targeted source control measures are recommended. First, strict implementation of special emission limits is essential to reduce atmospheric deposition. Second, irrigation water quality should be improved by decreasing suspended solids to minimize PTE input via water pathways. Additionally, straw removal may be considered to reduce PTE re-circulation and surface enrichment, provided that the removed straw is properly utilized to avoid secondary pollution while soil fertility is otherwise maintained.

Nevertheless, several inherent limitations introduce uncertainties into these long-term projections. The assumption of static 100-year flux baselines, omission of vertical leaching dynamics within a simplified homogeneous plow layer, and reliance on total instead of bioavailable concentrations collectively represent conservative, upper-bound estimations. Conversely, the exclusion of the dietary intake pathway via crop consumption suggests that actual cumulative risks may be underestimated. Future studies should incorporate dynamic scenario projections, bioaccessibility-based risk assessments, and dietary exposure modeling to provide a more comprehensive risk characterization. Overall, these findings demonstrate that regulatory compliance of soil does not inherently guarantee health safety related to soil exposure under the applied model and current scenarios. Spatially targeted interventions are imperative to mitigate cumulative, long-term soil exposure risks.

## Figures and Tables

**Table 1:** Soil Cd and As concentrations and pollution indices (PI, I_geo_, PLI) in the study areas.

Location	As (mg·kg^−1^)	Cd (mg·kg^−1^)	As_PI	Cd_PI	As_I_geo_	Cd_I_geo_	PLI
SK	10.10 ± 0.83	0.18 ± 0.06	0.85 ± 0.07	1.56 ± 0.52	−0.83 ± 0.12	0.04 ± 0.46	1.17
TG	10.21 ± 2.32	0.11 ± 0.03	0.86 ± 0.19	1.02 ± 0.29	−0.82 ± 0.33	−0.58 ± 0.42	0.94
HGZ	10.69 ± 1.05	0.12 ± 0.02	0.90 ± 0.09	1.03 ± 0.15	−0.75 ± 0.14	−0.55 ± 0.21	0.97
DB	9.66 ± 1.79	0.13 ± 0.08	0.81 ± 0.15	1.16 ± 0.70	−0.90 ± 0.26	−0.42 ± 0.87	0.98
Total	10.16 ± 1.55	0.13 ± 0.05	0.85 ± 0.13	1.18 ± 0.47	−0.83 ± 0.22	−0.39 ± 0.57	/
Background value	11.90	0.11	/	/	/	/	/
Screening value	20.00	0.30	/	/	/	/	/

Note: The background value originated from the background values of elements in China (1990); the standard value originated from GB15618-2018. The background value was used to calculate PI, I_geo_, and PLI. The standard value was used to determine whether the soil was polluted.

**Table 2:** The input fluxes of Cd and As in the study areas (g·ha^−1^·yr^−1^).

Element	Input Pathway	DB	HGZ	SK	TG
Cd	Atmospheric	0.69 ± 0.53	0.57 ± 0.36	1.24 ± 0.57	0.36 ± 0.15
	Irrigation water	0.40	1.19 ± 1.04	0.26 ± 0.12	0.30 ± 0.07
	Suspended solids	0.37 ± 0.09	0.34 ± 0.28	1.40 ± 2.56	0.30 ± 0.01
	Phosphate fertilizer	0.26	0.26	0.16	0.30
	Compound fertilizer	0.19	0.13	0.20	0.06
As	Atmospheric	29.43 ± 21.87	36.51 ± 30.26	36.86 ± 19.07	21.02 ± 7.72
	Irrigation water	14.08 ± 0.78	16.17 ± 8.37	13.64 ± 15.37	19.30 ± 20.03
	Suspended solids	21.06 ± 5.88	21.90 ± 17.29	80.54 ± 152.36	17.35 ± 0.31
	Phosphate fertilizer	9.22	8.89	5.46	10.40
	Compound fertilizer	9.79	6.77	10.49	3.37

**Table 3:** The output fluxes of Cd and As in the study areas (g·ha^−1^·yr^−1^).

Element	Output Pathway	DB	HGZ	SK	TG
Cd	Grain	0.03 ± 0.03	4.17 × 10^−3^	0.12 ± 0.12	0.43 ± 0.43
	Straw	0.90 ± 0.24	0.33 ± 0.07	0.42 ± 0.37	0.75 ± 0.47
	Leaching	0.23 ± 0.06	0.59 ± 0.72	0.35 ± 0.34	0.25 ± 0.21
As	Grain	0.51 ± 0.27	0.35 ± 0.14	0.41 ± 0.22	1.46 ± 1.57
	Straw	7.87 ± 0.85	6.51 ± 1.25	9.33 ± 5.77	19.27 ± 18.95
	Leaching	5.42 ± 0.67	10.15 ± 3.27	5.72 ± 3.20	13.13 ± 11.71

**Table 4:** The net fluxes under various scenarios (g·ha^−1^·yr^−1^).

Element	Study Area	Straw Off-Field	Straw Return
Cd	DB	0.75	1.65
	HGZ	1.56	1.89
	SK	2.36	2.79
	TG	−0.11	0.64
As	DB	69.78	77.65
	HGZ	73.22	79.73
	SK	131.53	140.86
	TG	37.57	56.84

## Data Availability

The raw data supporting the conclusions of this article will be made available by the authors on request.

## Supplementary Materials

The following supporting information can be downloaded at https://www.mdpi.com/article/10.3390/toxics14080652/s1. Table S1. Crop types and fertilizer applications at the actual soil sampling plots; Table S2. Analytical methods, instruments, and detection limits; Table S3. QA/QC summary; Table S4. The amount of dustfall and the content of Cd and As in the dustfall; Table S5. The contents of Cd and As in irrigation water and suspended solids; Table S6. The usage of irrigation water and the irrigation water utilization coefficient; Table S7. The concentrations of Cd and As in fertilizers (mg·kg^−1^); Table S8. The fertilizer application rate; Table S9. The concentration of Cd and As in different crop species and sections; Table S10. The yields of main crops; Table S11. The ratio of grain to straw of main crops; Table S12. The content of As and Cd in leachate; Table S13. The annual precipitation and evaporation; Table S14. Descriptive statistics of As and Cd concentrations and fluxes in various media; Table S15. Exposure parameters of the health risk assessment of soil; Table S16. Chronic reference dose (RfD) and slope factor (SF) values of Cd and As in soils in the health risk assessment; Table S17. Soil physicochemical properties, including pH, organic matter, and CEC; Table S18. The flux of atmospheric deposition in study areas (g·ha^−1^·yr^−1^); Table S19. The flux of irrigation in study areas (g·ha^−1^·yr^−1^); Table S20. The Cd and As flux of fertilizers in study areas (g·ha^−1^·yr^−1^); Table S21. The Cd and As flux of crop harvesting in study areas (g·ha^−1^·yr^−1^); Table S22. The Cd and As flux of leaching in study areas (g·ha^−1^·yr^−1^); Table S23. Back-calculation results for Cd and As. Figure S1. Cd exposure-pathway contributions to HI and TCR at year 100 for children, adults, and total risk; Figure S2. As exposure-pathway contributions to HI and TCR at year 100 for children, adults, and total risk [26,27,28,30,31,40,58,59,60,61,62,63,64].
