# Supplementary material for: Predicting Cadmium and Arsenic Accumulation and Soil-Exposure Health Risks in Agricultural Soils Below the Risk Screening Values: A Refined Flux Balance Model
Source: Toxics. 2026 Jul 24;14(8):652. doi: 10.3390/toxics14080652 (PMC13517913; doi:10.3390/toxics14080652)
Supplement: Supplementary file 1 [file toxics-14-00652-s001.zip › toxics-4342395-supplementary.pdf]

Table of contents

Table S1 Crop types and fertilizer applications at the actual soil sampling plots.

Table S2 Analytical methods, instruments, and detection limits.

Table S3 QA/QC summary.

Table S4 The amount of dustfall and the content of Cd and As in the dustfall.

Table S5 The contents of Cd and As in irrigation water and suspended solids.

Table S6 The usage of irrigation water and the irrigation water utilization coefficient.

Table S7 The concentrations of Cd and As in fertilizers ($mg \cdot kg^{-1}$).

Table S8 The fertilizer application rate.

Table S9 The concentration of Cd and As in different crop species and sections.

Table S10 The yields of main crops.

Table S11 The ratio of grain to straw of main crops.

Table S12 The content of As and Cd in leachate.

Table S13 The annual precipitation and evaporation.

Table S14 Descriptive statistics of As and Cd concentrations and fluxes in various media.

Table S15 Exposure parameters of the health risk assessment of soil.

Table S16 Chronic reference dose (RfD) and slope factor (SF) values of Cd and As in soils in the health risk assessment.

Table S17 Soil physicochemical properties, including pH, organic matter, and CEC.

Table S18 The flux of atmospheric deposition in study areas ($g \cdot ha^{-1} \cdot yr^{-1}$).

Table S19 The flux of irrigation in study areas ($g \cdot ha^{-1} \cdot yr^{-1}$).

Table S20 The Cd and As flux of fertilizers in study areas ($g \cdot ha^{-1} \cdot yr^{-1}$).

Table S21 The Cd and As flux of crop harvesting in study areas ($g \cdot ha^{-1} \cdot yr^{-1}$).

Table S22 The Cd and As flux of leaching in study areas ($g \cdot ha^{-1} \cdot yr^{-1}$).

Table S23 Back-calculation results for Cd and As.

Text S1 The calculation of Cd and As flux of fertilizers in study area.

The dominant agricultural inputs in these regions are compound fertilizer and urea. Single-nutrient phosphate and potassium fertilizers are rarely purchased or applied on-site; consequently, we were unable to obtain dedicated phosphate and potassium fertilizer samples. We collected nine fertilizer samples used by local farmers across four study sites and submitted them to a third-party laboratory for Cd and As analysis. The samples comprised urea and three types of compound fertilizer (NP, PK, and NPK). According to the officially stamped laboratory reports, Cd and As concentrations in urea were reported as zero (Table S7), which we interpret as being below the laboratory's reporting limit for these analytes in this matrix. This result is consistent with previous studies, which report that N fertilizers, including urea, generally have the lowest trace element concentrations among common fertilizer types, with most analyte concentrations below 1 $\mu g \cdot g^{-1}$ [1]. Because Cd and As in urea were below detection and therefore contribute negligibly to the total input flux, urea was excluded from subsequent flux calculations. For PK compound fertilizer, Cd was reported as zero (below detection), whereas As was detected. The detected As in PK compound fertilizer was attributed primarily to the phosphate-containing component rather than to the potassium component, since K fertilizers have also been reported to generally have low trace-element concentrations, with more than half of analytes below 1 $\mu g \cdot g^{-1}$ [1]. Consequently, we consider the Cd and As contribution from the K-fertilizer component to be negligible.

The input flux of phosphate fertilizer was not derived from a directly collected phosphate fertilizer sample, but was instead estimated using a proxy approach. Given that Cd and As in phosphate-containing fertilizers are known to originate primarily from impurities in the phosphate rock feedstock, and that phosphate fertilizers have been reported to contain substantially higher Cd and As concentrations than N or K fertilizers

[1], we used the measured Cd and As concentrations in NP compound fertilizer as a proxy for phosphate fertilizer, combined with the phosphate fertilizer application rates reported in the statistical yearbooks, to estimate the phosphate fertilizer input flux. Similarly, the measured concentrations in NPK compound fertilizer, combined with the compound fertilizer application rates from the statistical yearbooks, were used to estimate the compound fertilizer input flux.

Table S1 Crop types and fertilizer applications at the actual soil sampling plots.

Site ID	Location	Crop type	Fertilizer type
SS1	SK	Soybean	Urea, NPK compound fertilizer, NP compound fertilizer
SL1		Wheat	
SL2		Maize	
SL3		Maize	
SL4		Maize	
TS1	TG	Maize	Urea, compound fertilizer
TS2		Maize	
TS3		Rice	
TS4		Maize	
TS5		Rice	
HS1	HGZ	Maize	Urea, NP compound fertilizer
HS2		Maize	
HS3		Maize	
HS4		Maize	
DS1	DB	Maize	Urea, NPK compound fertilizer, NP compound fertilizer
DS2		Maize	
DS3		Maize	
DS4		Maize	

Table S2 Analytical methods, instruments, and detection limits.

No.	Category	Parameter	Testing Standard Method	Instrument Name and Model	Calibration Validity Date	Detection Limit
1	Irrigation water/Leachate	pH	Water quality—Determination of pH value—Electrode method (HJ 1147-2020)	Multi-parameter Water Quality Analyzer Multi 3630 IDS	2023.12.7	/
2	Irrigation water/Leachate	Cadmium	Graphite furnace atomic absorption spectrophotometric method for the determination of copper, lead, and cadmium (from Water *and Wastewater Monitoring and Analysis Methods* (4th Edition, Supplement), State Environmental Protection Administration, 2002)	Atomic Absorption Spectrophotometer AA-6880	2024.2.27	0.1 $\mu g \cdot L^{-1}$
3	Irrigation water/Leachate	Arsenic	Water quality—Determination of mercury, arsenic, selenium, bismuth, and antimony—Atomic fluorescence spectrometry (HJ 694-2014)	Atomic Fluorescence Spectrometer Kylin-S12	2024.6.5	0.3 $\mu g \cdot L^{-1}$
4	Irrigation	Suspended Solids	Water quality—Determination of suspended solids—Gravimetric method (GB 11901-89)	Electronic Balance FA224	2024.2.26	/
5	Soil/Sediment	Organic Matter	Soil testing—Part 6: Method for determination of soil organic matter (NY/T 1121.6-2006)	Burette	2026.2.26	/
6	Soil/Sediment	Cation Exchange Capacity (CEC)	Soil—Determination of cation exchange capacity (CEC)—Hexaminecobalt trichloride extraction-spectrophotometric method (HJ 889-2017)	Burette	2026.2.26	0.08 $cmol(+) \cdot kg^{-1}$
7	Dustfall	Cadmium	Solid waste—Determination of Lead and Cadmium—Graphite Furnace Atomic Absorption Spectrophotometry (HJ 787-2016)	Atomic Absorption Spectrophotometer AA-6880	2024.2.27	0.1 $mg \cdot kg^{-1}$
8	Dustfall	Arsenic	Solid Waste—Determination of Mercury, Arsenic, Selenium, Bismuth, and Antimony—Microwave Dissolution / Atomic Fluorescence Spectrometry (HJ 702-2014)	Atomic Fluorescence Spectrometer Kylin-S12	2024.6.5	0.1 $mg \cdot kg^{-1}$

No.	Category	Parameter	Testing Standard Method	Instrument Name and Model	Calibration Validity Date	Detection Limit
9	Dustfall	Total Dustfall	Ambient air—Determination of dustfall—Gravimetric method (HJ 1221-2021)	Electronic Balance ME55/02	2024.2.26	0.3 $t \cdot km^{-2}$
10	Crops	Cadmium	National food safety standard—Determination of cadmium in foods (GB 5009.15-2014)	Atomic Absorption Spectrophotometer AA-6880	2024.2.27	0.001 $mg \cdot kg^{-1}$
11	Crops	Arsenic	National food safety standard—Determination of total arsenic and inorganic arsenic in foods (GB 5009.11-2014)	Atomic Fluorescence Spectrometer Kylin-S12	2024.6.5	0.01 $mg \cdot kg^{-1}$
12	Fertilizer	Cadmium	Fertilizers and soil conditioners—Determination of arsenic, cadmium, chromium, lead, and mercury contents (GB/T 39229-2020 / ISO 17318:2015)	Inductively Coupled Plasma Optical Emission Spectrometer (ICP-OES) ICAP PRO	2024.8.17	/
13	Fertilizer	Arsenic	Fertilizers and soil conditioners—Determination of arsenic, cadmium, chromium, lead, and mercury contents (GB/T 39229-2020 / ISO 17318:2015)	Inductively Coupled Plasma Optical Emission Spectrometer (ICP-OES) ICAP PRO	2024.6.5	/

Table S3 QA/QC summary.

Category	Index	N	Total program blank	Laboratory blank	Duplicate sample	External duplicate	Spike recovery sample	Certified reference material (CRM)	Qualified rate
Irrigation water	As	42	/	2	3	/	/	3	100
Irrigation water	Cd	42	/	2	3	/	/	3	100
Irrigation water	Suspended solids	40	/	/	5	/	/	/	100
Leachate	As	25	4	4	4	4	/	6	100
Leachate	Cd	25	4	4	4	4	2	4	100
Sediment	As	19	/	2	2	/	/	2	100
Sediment	Cd	19	/	2	2	/	/	2	100
Sediment	pH	33	/	/	4	/	/	4	100
Soil	As	33	/	4	5	/	/	5	100
Soil	Cd	33	/	4	5	/	/	5	100
Soil	pH	33	/	/	4	/	/	4	100
Soil	OM	33	/	2	4	/	/	4	100
Soil	CEC	33	/	2	4	/	/	4	100

Note: A total of 33 samples were reported in the detection report, but only 18 surface samples were selected for flux model calculations.

Table S4 The amount of dustfall and the content of Cd and As in the dustfall.

Location		As	Cd	Dustfall
		mg·kg^{-1}		t·km^{-2}, (30 d)$^{-1}$
HGZ	Max	40.00	0.78	40.20
	Min	21.00	0.27	4.40
	Average	29.19	0.51	11.83
	Std.	6.40	0.20	12.83
TG	Max	37.20	0.85	11.80
	Min	18.70	0.27	3.90
	Average	26.33	0.46	6.80
	Std.	6.00	0.19	2.77
SK	Max	30.10	1.62	18.20
	Min	20.40	0.34	5.10
	Average	24.16	0.92	12.46
	Std.	3.98	0.47	5.63
DB	Max	29.30	1.30	26.60
	Min	19.70	0.40	5.70
	Average	24.20	0.58	9.93
	Std.	3.40	0.32	7.57

Table S5 The contents of Cd and As in irrigation water and suspended solids.

Location		Cd and As in irrigation water		Content of suspended solids	Cd and As in suspended solids	
		As	Cd		As	Cd
		$mg \cdot L^{-1}$		$mg \cdot L^{-1}$	$mg \cdot kg^{-1}$	
TG	Max	9.30E-03	7.50E-05	445.50	9.71	0.16
	Min	1.70E-03	5.00E-05	342.50	7.20	0.13
	Average	3.64E-03	5.63E-05	402.17	8.24	0.14
	Std.	3.78E-03	1.25E-05	53.41	1.31	0.02
HGZ	Max	4.80E-03	4.75E-04	852.00	9.34	0.14
	Min	1.60E-03	5.00E-05	70.00	6.72	0.12
	Average	2.89E-03	2.13E-04	487.75	8.50	0.13
	Std.	1.50E-03	1.85E-04	400.87	1.20	0.01
DB	Max	1.90E-03	5.00E-05	419.50	8.13	0.14
	Min	1.60E-03	5.00E-05	195.00	6.29	0.12
	Average	1.75E-03	5.00E-05	353.25	7.32	0.13
	Std.	9.64E-05	0.00E+00	83.62	0.61	0.01
SK	Max	1.14E-02	1.25E-04	11134.00	9.34	0.16
	Min	1.75E-03	5.00E-05	353.50	6.48	0.13
	Average	3.21E-03	6.07E-05	2341.92	7.45	0.14
	Std.	3.61E-03	2.83E-05	4311.04	1.16	0.01

Table S6 The usage of irrigation water and the irrigation water utilization coefficient.

Location	The usage of irrigation water $m^3 \cdot m^{-2}$	Irrigation water utilization coefficient
TG	0.988	0.537
HGZ	1.042	0.537
DB	1.379	0.573
SK	0.765	0.566

Notes: Data from the Water Resources Bulletin of Ningxia Province in 2021 [2].

Table S7 The concentrations of Cd and As in fertilizers ($mg \cdot kg^{-1}$).

Location	Fertilizer type	As ($mg \cdot kg^{-1}$)	Cd ($mg \cdot kg^{-1}$)
DB	Urea	0	0
TG	Urea	0	0
DB	NP compound fertilizer	31.30	0.88
DB	PK compound fertilizer	13.60	0
HGZ	NPK compound fertilizer	21.40	0.40
SK	Urea	0	0
SK	PK compound fertilizer	13.80	0
TG	NPK compound fertilizer	14.70	0.29
SK	NP compound fertilizer	36.90	1.08

Note: A reported value of '0' indicates that the analyte was reported as not detected by the testing laboratory; no explicit method detection limit was provided in the original report for these fertilizer samples.

Table S8 The fertilizer application rate.

Location	Phosphate fertilizer	Compound fertilizer
	kg·m^{-2}	
DB	0.027	0.054
TG	0.030	0.019
HGZ	0.026	0.038
SK	0.016	0.058

Note: Data from Ningxia Statistical Yearbook (2021) [3].

Table S9 The concentration of Cd and As in different crop species and sections (mg·kg^{-1}).

Section		Type	Min	Max	Average	Std
Grain	Cd	Rice	0.002	0.085	0.044	0.059
		Maize	0.002	0.122	0.025	0.041
		Soybean	0.087	0.087	0.087	/
		Wheat	0.058	0.058	0.058	/
	As	Rice	0.210	0.510	0.360	0.210
		Maize	0.026	0.092	0.050	0.018
		Soybean	0.071	0.071	0.071	/
		Wheat	0.112	0.112	0.112	/
Straw	Cd	Rice	0.028	0.100	0.064	0.051
		Maize	0.022	0.128	0.054	0.031
		Soybean	0.159	0.159	0.159	/
		Wheat	0.105	0.105	0.105	/
	As	Rice	3.720	3.746	3.733	0.018
		Maize	0.430	1.200	0.690	0.220
		Soybean	0.702	0.702	0.702	/
		Wheat	0.607	0.607	0.607	/

Note: “/” indicates that the standard deviation was not calculable due to a single measurement (n = 1).

Table S10 The yields of main crops (t·ha^{-1}).

Section	Location	Rice	Maize	Soybean	Wheat
Grain	DB	9.34	9.90	2.15	5.74
	TG	8.10	8.56	/	5.10
	HGZ	6.64	8.34	0.93	5.57
	SK	9.00	9.87	3.26	3.37
Straw	DB	12.36	12.56	3.43	9.86
	TG	10.72	10.87	/	8.75
	HGZ	8.79	10.58	1.48	9.57
	SK	11.91	12.52	5.22	5.79

Note: Data from Ningxia Statistical Yearbook (2021) [3].

Table S11 The ratio of grain to straw of main crops.

Type	Ratio of grain to straw
Rice	0.756
Maize	0.788
Soybean	0.625
Wheat	0.582

Note: Data from Zhang and Zhu [4]

Table S12 The content of As and Cd in leachate.

Location		As	Cd
		mg·L^{-1}	
SK	Max	3.70E-03	2.75E-04
	Min	1.60E-03	5.00E-05
	Average	2.65E-03	1.63E-04
	Std.	1.48E-03	1.59E-04
DB	Max	1.50E-03	7.50E-05
	Min	1.20E-03	5.00E-05
	Average	1.40E-03	5.83E-05
	Std.	1.73E-04	1.44E-05
HGZ	Max	3.50E-03	4.00E-04
	Min	1.80E-03	5.00E-05
	Average	2.85E-03	1.67E-04
	Std.	9.18E-04	2.02E-04
TG	Max	1.04E-02	2.00E-04
	Min	9.00E-04	5.00E-05
	Average	4.26E-03	8.00E-05
	Std.	3.80E-03	6.71E-05

Table S13 The annual precipitation and evaporation.

Location	Annual Precipitation	Annual Evaporation
	mm·yr^{-1}	
SK	198	322
DB	159	347
HGZ	137	263
TG	173	322

Note: Data from the Ningxia Meteorological Bureau.

Table S14 Descriptive statistics of As and Cd concentrations and fluxes in various media.

Variable	n	Mean ± SD	95% CI	Median	Min – Max	CV (%)
Soil As concentration ($mg\cdot kg^{-1}$)	18	10.21 ± 1.52	9.45 − 10.97	10.55	7.42 – 13.50	14.89
Soil Cd concentration ($mg\cdot kg^{-1}$)	18	0.13 ± 0.05	0.11 − 0.16	0.12	0.07 – 0.24	38.10
Atmospheric As input flux ($g\cdot ha^{-1}\cdot yr^{-1}$)	26	30.50 ± 21.23	21.93 − 39.08	22.01	13.10 – 102.71	69.60
Atmospheric Cd input flux ($g\cdot ha^{-1}\cdot yr^{-1}$)	26	0.67 ± 0.50	0.47 – 0.87	0.42	0.20 – 1.86	74.52
Compound fertilizer As input flux ($g\cdot ha^{-1}\cdot yr^{-1}$)	4	7.61 ± 3.25	2.43 – 12.78	8.28	3.37 – 10.49	42.76
Compound fertilizer Cd input flux ($g\cdot ha^{-1}\cdot yr^{-1}$)	4	0.15 ± 0.06	0.04 – 0.25	0.16	0.06 – 0.20	44.48
Phosphate fertilizer As input flux ($g\cdot ha^{-1}\cdot yr^{-1}$)	4	8.49 ± 2.12	5.11 – 11.87	9.06	5.46 – 10.40	24.99
Phosphate fertilizer Cd input flux ($g\cdot ha^{-1}\cdot yr^{-1}$)	4	0.25 ± 0.06	0.15 – 0.34	0.26	0.16 – 0.30	24.49
Irrigation supernatant As concentration ($mg\cdot L^{-1}$)	23	2.72E-03 ± 2.53E-03	1.63E-03 – 3.81E-03	1.80E-03	1.60E-03 – 1.14E-02	92.93
Irrigation supernatant As flux ($g\cdot ha^{-1}\cdot yr^{-1}$)	23	15.22 ± 11.55	10.22 – 20.21	12.87	7.44 – 49.35	75.89

Variable	n	Mean ± SD	95% CI	Median	Min – Max	CV (%)
Irrigation supernatant Cd concentration (mg·L^{-1})	23	8.26E-05 ± 9.31E-05	4.24E-05 – 1.23E-04	5.00E-05	5.00E-05 – 4.75E-04	112.66
Irrigation supernatant Cd flux (g·ha^{-1}·yr^{-1})	23	0.48 ± 0.52	0.25 – 0.70	0.40	0.21 – 2.66	108.23
Irrigation suspended-solid As flux (g·ha^{-1}·yr^{-1})	19	39.44 ± 85.63	-1.84 – 80.71	20.12	3.52 – 391.17	217.15
Irrigation suspended-solid Cd flux (g·ha^{-1}·yr^{-1})	19	0.68 ± 1.45	-0.02 – 1.38	0.39	0.05 – 6.63	213.79
Suspended-solid As concentration (mg·kg^{-1})	19	7.75 ± 1.08	7.24 – 8.27	7.51	6.29 – 9.71	13.88
Suspended-solid Cd concentration (mg·kg^{-1})	19	0.13 ± 0.01	0.13 – 0.14	0.13	0.12 – 0.16	8.70
Suspended-solid concentration, SSC (mg·L^{-1})	19	1017.29 ± 2459.43	-168.16 – 2202.74	404.50	70.00 − 11134.00	241.76
Leachate As concentration (mg·L^{-1})	13	3.03E-03 ± 2.54E-03	1.49E-03 – 4.56E-03	1.80E-03	9.00E-04 − 1.04E-02	84.01
Leachate Cd concentration (mg·L^{-1})	13	1.08E-04 ± 1.13E-04	3.95E-05 – 1.76E-04	5.00E-05	5.00E-05 − 4.00E-04	104.84
Leaching As output flux (g·ha^{-1}·yr^{-1})	13	1.59 ± 1.30	0.80 – 2.37	1.07	0.46 − 5.34	81.71

**Variable**	**n**	**Mean ± SD**	**95% CI**	**Median**	**Min – Max**	**CV (%)**
Leaching Cd output flux ($g·ha^{-1}·yr^{-1}$)	13	0.06 ± 0.06	0.02 – 0.09	0.03	0.02 − 0.24	108.31
Grain As concentration ($mg·kg^{-1}$)	18	0.09 ± 0.11	0.03 – 0.14	0.05	0.03 − 0.51	127.79
Grain As removal flux ($g·ha^{-1}·yr^{-1}$)	18	0.71 ± 0.92	0.26 – 1.17	0.46	0.07 − 4.12	128.62
Grain Cd concentration ($mg·kg^{-1}$)	18	0.02 ± 0.04	0.01 – 0.04	0.01	0.00 − 0.12	156.44
Grain Cd removal flux ($g·ha^{-1}·yr^{-1}$)	18	0.16 ± 0.28	0.02 – 0.30	0.05	0.00 − 1.04	175.51
Straw As concentration ($mg·kg^{-1}$)	18	1.02 ± 1.01	0.52 – 1.52	0.65	0.43 − 3.75	98.24
Straw As removal flux ($g·ha^{-1}·yr^{-1}$)	18	11.14 ± 10.99	5.68 – 16.60	7.14	1.04 − 40.16	98.63
Straw Cd concentration ($mg·kg^{-1}$)	18	0.06 ± 0.04	0.04 – 0.08	0.05	0.00 − 0.16	68.86
Straw Cd removal flux ($g·ha^{-1}·yr^{-1}$)	18	0.60 ± 0.39	0.41 – 0.79	0.43	0.01 − 1.39	64.52

Note: The 95% CI can fall below zero because of the limitations of symmetric interval approximation for small and highly skewed samples; such bounds are not physically meaningful.

Table S15 Exposure parameters of the health risk assessment of soil.

Parameter	Description	Unit	Children	Adults	Reference
IR_{ing}	Soil ingestion rate	$mg \cdot d^{-1}$	200	100	[5-7]
EF	Exposure frequency	$d \cdot yr^{-1}$	350	350	[5-7]
ED	Exposure duration	yr	6	24	[5-7]
IR_{inh}	Inhalation rate	$m^3 \cdot d^{-1}$	7.6	20	[8]
PEF	Particle emission factor	$m^3 \cdot kg^{-1}$	1.36E+09	1.36E+09	[6]
BW	Average body weight	kg	19.2	61.8	[5]
AT_{HI}	Averaging time for non-carcinogenic risk (HI)	d	2190	8760	[6,7]
AT_{CR}	Averaging time for carcinogenic risk (CR)	d	25550	25550	[6]
SA	Exposed skin area	cm^2	2800	5700	[7]
AF	Skin adherence factor	$mg \cdot cm^{-2}$	0.2	0.07	[5,7,9]
ABF	Dermal adsorption factor	-	0.001	0.001	[10]

Table S16 Chronic reference dose (RfD) and slope factor (SF) values of Cd and As in soils in health risk assessment.

Parameter	Route	As	Cd
RfD ($mg \cdot kg^{-1} \cdot d^{-1}$)	ingestion	3.00E-04 [11]	1.00E-03 [13]
	inhalation	1.23E-04 [9]	1.00E-05 [13]
	dermal	1.23E-04 [9]	1.00E-05 [13]
SF ($kg \cdot d \cdot mg^{-1}$)	ingestion	1.50E+00 [11]	6.10E+00 [11]
	inhalation	1.51E+01 [12]	6.30E+00 [13]
	dermal	3.66E+00 [12]	-

Table S17 Soil physicochemical properties, including pH, organic matter, and CEC.

Soil property	Unit	Min	Median	Max	Average	Std
pH	/	8.06	8.48	8.79	8.48	0.20
OM	g·kg-1	12.60	15.50	23.50	16.64	3.48
CEC	cmol(+)·kg-1	6.20	9.60	12.20	9.52	1.72

Table S18 The flux of atmospheric deposition in study areas (g·ha^{-1}·yr^{-1}).

Location	Cd					As				
	Min	Median	Max	Average	Std	Min	Median	Max	Average	Std
DB	0.28	0.37	1.52	0.69	0.53	13.66	18.03	75.41	29.43	21.87
HGZ	0.28	0.44	1.32	0.57	0.36	15.26	27.25	102.71	36.51	30.26
SK	0.52	1.45	1.86	1.24	0.57	16.20	35.49	60.79	36.86	19.07
TG	0.20	0.30	0.67	0.36	0.15	13.10	20.54	36.61	21.02	7.72
Total	0.20	0.42	1.86	0.67	0.50	13.10	22.00	102.71	30.50	21.20

Table S19 The flux of irrigation in study areas (g·ha^{-1}·yr^{-1}).

Type	Element	Study Area	Min	Median	Max	Average	Std
Supernatant from irrigation water	Cd	DB	0.40	0.40	0.40	0.40	0.00
		HGZ	0.28	0.91	2.66	1.19	1.04
		SK	0.21	0.21	0.53	0.26	0.12
		TG	0.27	0.27	0.40	0.30	0.07
	As	DB	12.87	14.28	15.29	14.08	0.78
		HGZ	8.96	14.42	26.87	16.17	8.37
		SK	7.44	7.87	48.49	13.64	15.37
		TG	9.02	9.42	49.35	19.30	20.03
Suspended solids of irrigation water	Cd	DB	0.19	0.41	0.42	0.37	0.09
		HGZ	0.05	0.37	0.59	0.34	0.28
		SK	0.20	0.41	6.63	1.40	2.56
		TG	0.29	0.29	0.31	0.30	0.01
	As	DB	9.87	22.63	26.26	21.06	5.88
		HGZ	3.52	21.78	40.54	21.90	17.29
		SK	9.79	19.36	391.17	80.54	152.36
		TG	17.02	17.37	17.65	17.35	0.31

Table S20 The Cd and As flux of fertilizers in study areas (g·ha^{-1}·yr^{-1}).

Location	Phosphate fertilizer		Compound fertilizer		Total	
	As	Cd	As	Cd	As	Cd
DB	9.22	0.26	9.79	0.19	19.01	0.45
HGZ	8.89	0.26	6.77	0.13	15.66	0.38
SK	5.46	0.16	10.49	0.20	15.95	0.36
TG	10.40	0.30	3.37	0.06	13.76	0.36

Table S21 The Cd and As flux of crop harvesting in study areas (g·ha^{-1}·yr^{-1}).

Section	Element	Study Area	Min	Median	Max	Average	Std
Grain	Cd	DB	4.95E-03	2.48E-02	7.92E-02	3.34E-02	3.22E-02
		HGZ	4.17E-03	4.17E-03	4.17E-03	4.17E-03	0.00
		SK	4.93E-03	8.88E-02	0.32	0.12	0.12
		TG	1.62E-02	0.33	1.04	0.43	0.43
	As	DB	0.32	0.41	0.91	0.51	0.27
		HGZ	0.22	0.33	0.55	0.35	0.14
		SK	0.07	0.46	0.62	0.41	0.22
		TG	0.43	0.57	4.12	1.46	1.57
Straw	Cd	DB	0.69	0.85	1.22	0.90	0.24
		HGZ	0.23	0.35	0.39	0.33	0.07
		SK	0.01	0.39	1.01	0.42	0.37
		TG	0.30	0.61	1.39	0.75	0.47
	As	DB	7.07	7.88	8.66	7.87	0.85
		HGZ	5.24	6.30	8.19	6.51	1.25
		SK	1.04	11.61	15.03	9.33	5.77
		TG	4.69	6.19	40.16	19.27	18.95

Table S22 The Cd and As flux of leaching in study areas (g·ha^{-1}·yr^{-1}).

Element	Study Area	Min	Median	Max	Average	Std
Cd	DB	0.19	0.19	0.29	0.23	0.06
	HGZ	0.18	0.18	1.43	0.59	0.72
	SK	0.11	0.35	0.59	0.35	0.34
	TG	0.15	0.15	0.62	0.25	0.21
As	DB	4.65	5.81	5.81	5.42	0.67
	HGZ	6.41	11.58	12.47	10.15	3.27
	SK	3.45	5.72	7.98	5.72	3.20
	TG	2.77	10.79	32.05	13.13	11.71

Table S23 Back-calculation results for Cd and As.

Element	Region	Current ($mg \cdot kg^{-1}$)	Background ($mg \cdot kg^{-1}$)	ΔF ($g \cdot ha^{-1} \cdot yr^{-1}$)	Years from background
Cd	DB	0.13	0.112	0.75	55
	HGZ	0.12	0.112	1.56	12
	SK	0.18	0.112	2.36	66
	TG	0.11	0.112	−0.11	N/A—below background
As	DB	9.66	11.9	69.78	74
	HGZ	10.69	11.9	73.22	38
	SK	10.10	11.9	131.53	31
	TG	10.21	11.9	37.57	103

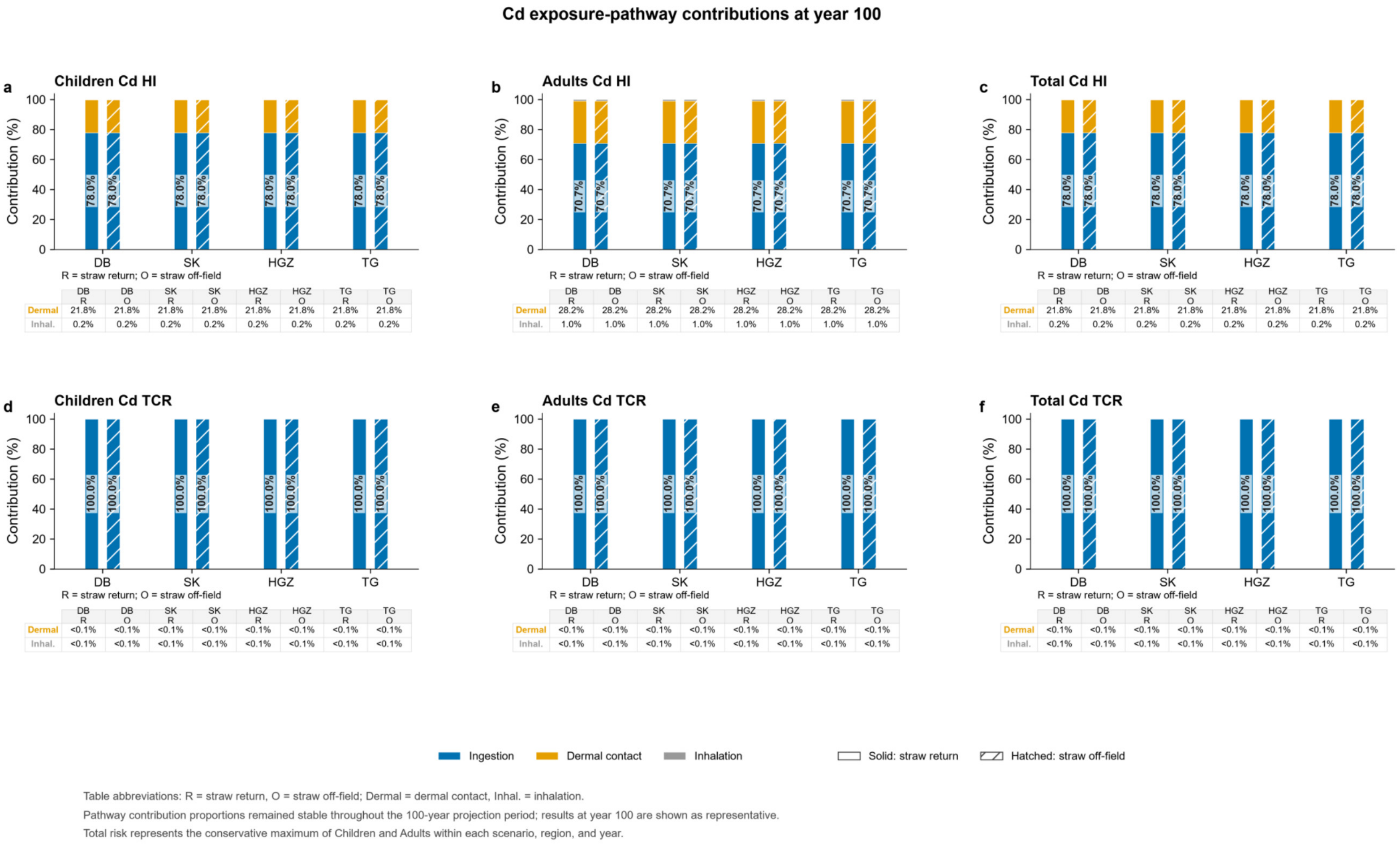

Figure S1. Cd exposure-pathway contributions to HI and TCR at year 100 for children, adults, and total risk.

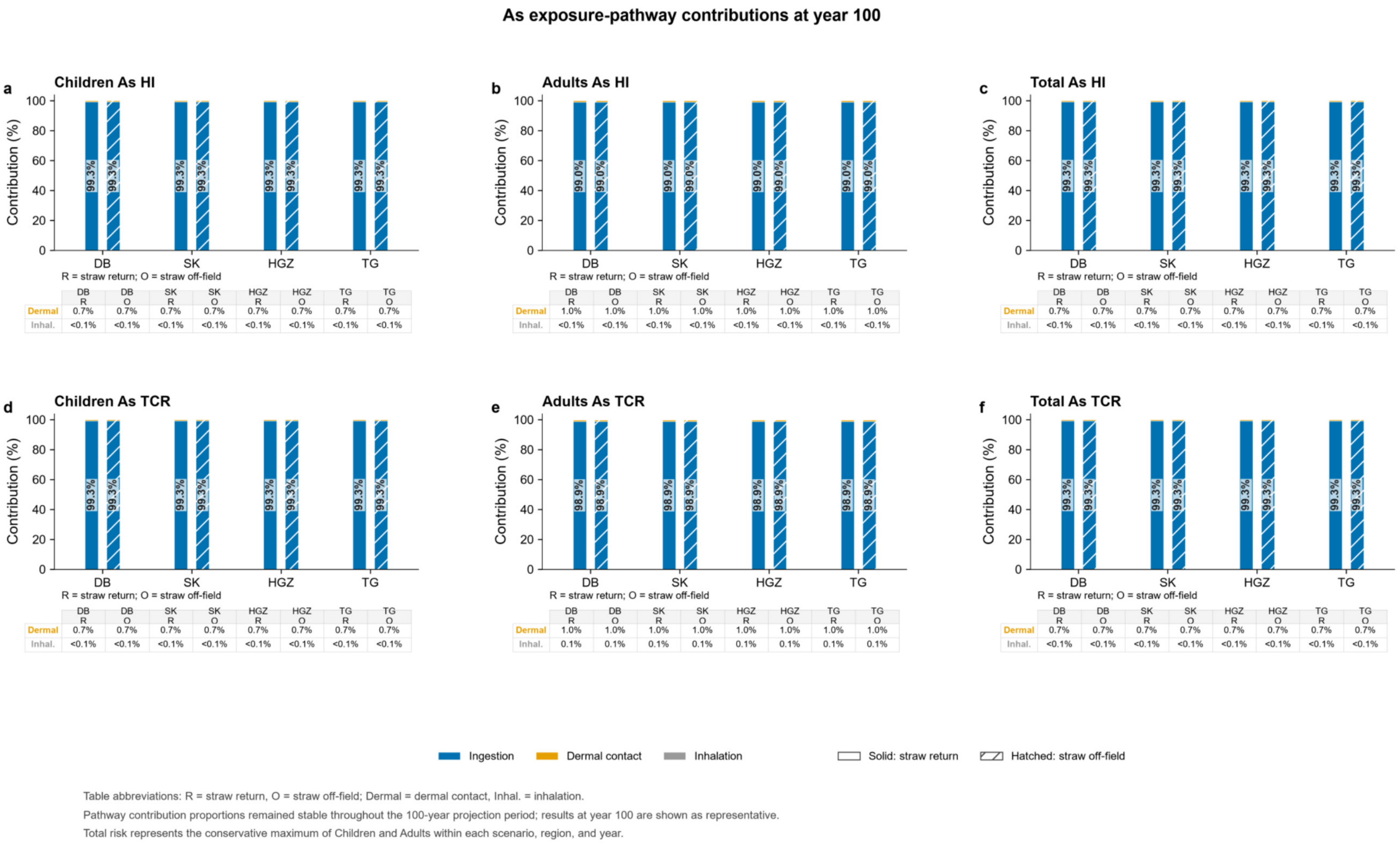

Figure S2. As exposure-pathway contributions to HI and TCR at year 100 for children, adults, and total risk.

References

1. Raven, K.P.; Loeppert, R.H. Trace element composition of fertilizers and soil amendments. *J. Environ. Qual.* **1997**, *26*(2), 551–557. https://doi.org/10.2134/jeq1997.00472425002600020028x.
2. Ningxia Hui Autonomous Region Water Resources Department; Ningxia Hydrology and Water Resources Monitoring and Early Warning Center. *The Water Resources Bulletin of Ningxia Province in 2021*; Ningxia Hui Autonomous Region Water Resources Department and Ningxia Hydrology and Water Resources Monitoring and Early Warning Center: Yinchuan, China, 2022.
3. Statistics Bureau of Ningxia Hui Autonomous Region; Ningxia Investigation Team of National Bureau of Statistics. *Ningxia Statistical Yearbook 2021*; China Statistics Press Co., Ltd.: Beijing, China, **2021**.
4. Zhang, F.; Zhu, Z. Harvest index for various crops in China. Sci. Agric. Sin. **1990**, 2, 83–87.
5. Ministry of Ecology and Environment. Technical Guidelines for Risk Assessment of Soil Contamination of Land for Construction: HJ 25.3-**2019**; China Environment Publishing Group: Beijing, China, **2019**.
6. U.S. Environmental Protection Agency (U.S. EPA). Exposure Factors Handbook: 2011 edition; National Center for Environmental Assessment: Washington, DC, USA, **2011**.
7. Miletić, A.; Lučić, M.; Onjia, A. Exposure factors in health risk assessment of heavy metal(loid)s in soil and sediment. *Metals* **2023**, *13*(7), 1266. https://doi.org/10.3390/met13071266.
8. Xu, Y.; Dai, S.; Meng, K.; Wang, Y.; Ren, W.; Zhao, L.; Christie, P.; Teng, Y. Occurrence and risk assessment of potentially toxic elements and typical organic pollutants in contaminated rural soils. *Sci. Total Environ.* **2018**, *630*, 618–629. https://doi.org/10.1016/j.scitotenv.2018.02.212.
9. Chen, R.; Chen, H.; Song, L.; Yao, Z.; Meng, F.; Teng, Y. Characterization and source apportionment of heavy metals in the sediments of Lake Tai (China) and its surrounding soils. *Sci. Total Environ.* **2019**, *694*, 133819. https://doi.org/10.1016/j.scitotenv.2019.133819.
10. U.S. Environmental Protection Agency (U.S. EPA). Risk Assessment Guidance for Superfund, Volume I: Human Health Evaluation Manual (Part E, Supplemental Guidance for Dermal Risk Assessment); Office of Emergency and Remedial Response: Washington, DC, USA, **2004**.
11. Yang, S.; Zhao, J.; Chang, S.X.; Collins, C.; Xu, J.; Liu, X. Status assessment and probabilistic health risk modeling of metals accumulation in agriculture soils across China: A synthesis. *Environ. Int.* **2019**, *128*, 165–174. https://doi.org/10.1016/j.envint.2019.04.044.
12. Han, Q.; Wang, M.; Cao, J.; Gui, C.; Liu, Y.; He, X.; He, Y.; Liu, Y. Health risk assessment and bioaccessibilities of heavy metals for children in soil and dust from urban parks and schools of Jiaozuo, China. *Ecotoxicol. Environ. Saf.* **2020**, *191*, 110157. https://doi.org/10.1016/j.ecoenv.2019.110157.
13. Gu, Y.G.; Lin, Q.; Gao, Y.P. Metals in exposed-lawn soils from 18 urban parks and its human health implications in southern China's largest city, Guangzhou. *J. Clean. Prod.* **2016**, *115*, 122–129. https://doi.org/10.1016/j.jclepro.2015.12.031.
